# Mindfulness-based interventions for binge eating: an updated systematic review and meta-analysis

**DOI:** 10.1007/s10865-025-00550-5

**Published:** 2025-02-20

**Authors:** Jianyi Liu, Mara Tynan, Alexandra Mouangue, Caroline Martin, Stephanie Manasse, Kathryn Godfrey

**Affiliations:** 1 Department of Psychological and Brain Sciences, Drexel University, Stratton Hall, 3201 Chestnut Street, Philadelphia, PA 19104 USA; 2 Center for Weight, Eating and Lifestyle Science, Drexel University, Philadelphia, PA USA; 3 Joint Doctoral Program in Clinical Psychology, San Diego State University, University of California, San Diego, CA USA; 4 Center for WorkLife Wellbeing, ChristianaCare, Wilmington, DE USA

**Keywords:** Mindfulness, Binge eating, Dialectical behavior therapy, Acceptance and commitment therapy

## Abstract

Mindfulness-based interventions (MBIs) have gained popularity in recent years in treating binge eating. Previous reviews and meta-analyses have found that MBIs demonstrated medium-large to large effects in reducing binge eating. However, as the literature on this topic has been growing rapidly, an updated review on MBIs’ effectiveness is much needed. This study is a 10-year update of the Godfrey, Gallo, & Afari (2015) systematic review and meta-analysis of MBIs for binge eating. PubMED, PsycINFO, and Web of Science were searched using keywords including binge eating, overeating, objective bulimic episodes, acceptance and commitment therapy, dialectical behavior therapy, mindfulness, meditation, and mindful eating. Results indicate there has been a large increase in the number of studies testing MBIs for binge eating in the past 10 years with 54 studies meeting inclusion criteria, compared to 19 ten years ago. The majority of the studies yielded large and medium effect sizes. The random effects meta-analysis of between-group effect sizes yielded medium-large effects for MBIs versus non-psychological intervention controls at post-treatment (mean Hedge’s *g* = − 0.65) and follow-up (mean Hedge’s *g* = − 0.71), and negligible effects for MBIs versus active psychological controls at post-treatment (mean Hedge’s *g* = − 0.05) and follow-up (mean Hedge’s *g* = 0.13). Of all MBIs, DBT had the most studies with large effects. More studies examined MBIs that directly targeted binge eating had larger effects than studies with MBIs targeting other health outcomes (with binge eating as a secondary outcome). New studies included in the current review were internationally-conducted, focused more on participants with overweight or obesity, involved more self-help and technology-based components, and had more novel and innovative interventions components. Future MBIs research should conduct more RCTs comparing MBIs with other psychological interventions, conduct meta-analyses to examine the effectiveness of different types of MBIs and intervention targets, and extend follow-up periods.

## Introduction

Binge eating is characterized by eating a large amount of food within a discrete period of time, accompanied by a sense of loss of control over eating (American Psychiatric Association, 2022). Over 42 million Americans are estimated to struggle with clinically significant binge eating (i.e., binge eating at least once per week over the past three months; Mitchison et al., 2017), which is concerning given its association with physical health consequences (e.g., diabetes and cardiovascular disease; Kessler et al., 2013) and psychological comorbidities (e.g., depression, anxiety, and substance abuse; Bogusz et al., 2021; Pisetsky et al., 2016; Udo & Grilo, 2018). Cognitive-behavioral therapy (CBT) is currently the gold-standard treatment for patients with binge eating (Fairburn et al., 2009). However, research has shown that only 30% of patients achieve full remission (i.e., cessation of binge eating) at the end of the treatment (Linardon & Wade, 2018). Moreover, over 30% of patients who achieve remission relapse within 12 months of completing CBT (Södersten et al., 2017). There is an urgent need to develop and test innovative treatment strategies to further improve treatment outcomes for patients with binge eating.

Mindfulness-based interventions (MBIs), including acceptance and commitment therapy (ACT), dialectical behavior therapy (DBT), mindfulness-based stress reduction (MBSR), and other interventions using mindfulness strategies, have become increasingly popular in recent years (Grohmann & Laws, 2021). These interventions typically involve having patients practice mindfulness strategies to increase awareness and acceptance of their internal experiences, build distress tolerance, and decrease avoidance behaviors (Baer et al., 2005b). Poor ability to cope with negative emotions and tendency to avoid unpleasant experiences have been demonstrated as core maintaining factors of binge eating (Van Strien et al., 2005). Therefore, MBIs could be particularly helpful in breaking this cycle by teaching patients to attend to internal experiences in the present moment in a non-judgmental way (Bishop et al., 2004), which could help them form new adaptive coping skills (Mallorqui-Bague et al., 2018) and strengthen their interoceptive awareness (Gibson, 2019) and cognitive control (Zeidan et al., 2010).

Godfrey & colleagues (2015) conducted a systematic review and meta-analysis on MBIs on binge eating, including both randomized control trials (RCTs) and uncontrolled cohort studies (UCSs). Studies included in Godfrey et al.’s review (2015) included samples who engaged in objective bulimic episodes (OBEs) and bariatric samples who engaged in subjective bulimic episodes (SBEs). The authors found a large within-group effect size for MBIs in reducing binge eating (Hedge’s g = 1.12, [95% CI − 1.67, − 0.80]). The authors also reported a large between-group effect size for RCTs comparing MBIs with control groups, including waitlists and treatment as usual (TAU; Hedge’s g = 0.70 [95% CI 1.16, 0.24]). However, the authors reported that the quality of evidence was limited due to selection bias and high dropout rates. A more recent systematic review and meta-analysis on MBIs for binge eating was conducted by Grohmann and Laws (2021). In this review, Grohmann and Laws (2021) also included studies where the inclusion criteria for binge episodes did not include the characteristic of being objectively large in sizes. The authors also examined the impact of MBIs on various psychological symptoms, such as anxiety, depression, and psychological well-being. Grohmann and Laws reported a small effect size for MBIs in reducing binge eating at the end of the treatment (Hedge’s g = − 0.39 [95% CI − 0.68, − 0.11]), and a close-to-zero effect size at follow-up (Hedge’s g = − 0.06 [95% CI − 0.31, 0.20]). The authors reported that the low effect size at follow-up could be due to the small number of studies that reported follow-up data. Additionally, the authors found that the effect size was larger for comparisons with waitlist controls (Hedge’s g = − 0.73 [95% CI − 1.09, − 0.37]) versus active controls (Hedge’s g = − 0.15, [95% CI − 0.96, 0.34]). The review by Grohmann and Laws (2021) also included studies that only examined constructs similar to binge eating (e.g., emotional eating) as well as studies that did not directly measure binge eating as an outcome (e.g., only reported global eating pathology). Thus, an updated systematic review more tightly focusing on binge eating could help researchers and clinicians better understand MBIs’ effectiveness for this specific population. Therefore, an updated systematic review and meta-analysis are needed to further assess the development in this fast-growing field.

The current review and meta-analysis is an update of Godfrey et al.’s (2015) review of MBIs for binge eating. The current review’s aim is to summarize and synthesize the existing research efforts in examining the effectiveness of MBIs for binge eating. This systematic review includes RCTs that compared changes in binge eating in patients receiving MBIs with control groups, waitlists, or treatment as usual, and UCSs that examined improvement in binge eating after receiving MBIs in single groups. This review and meta-analysis describe the studies’ characteristics, examine the overall evidence for the effectiveness of these interventions in reducing binge eating, examine changes in the literature since the original review, and discuss remaining opportunities to advance the research on MBIs for binge eating.

## Methods

### Search strategy

The current review replicated the methods used by Godfrey et al. (2015). The Preferred Reporting Items for Systematic Reviews and Meta-Analyses (PRISMA) guidelines were used for this systematic review (Liberati et al., 2009). PubMED (from 1953 to July 23, 2024), PsycINFO (from 1806 to July 23, 2024), and Web of Science (from 1900 to July 23, 2024) were searched using the following terms: binge eating OR binge eating disorder OR overeating OR objective bulimic episodes AND acceptance and commitment therapy OR dialectical behavior therapy OR mindfulness OR meditation OR mindful eating. Listservs associated with the American Psychological Association’s Division 38 (Health Psychology), Society of Behavioral Medicine, Association for Behavioral and Cognitive Therapies, and Association for Contextual Behavioral Science were used to collect unpublished data to minimize publication bias. The National Institutes of Health’s clinicaltrial.gov was searched for relevant studies.

### Inclusion and exclusion criteria

Studies were included in the screening process if they were written in English, published in peer-reviewed scholarly journals, RCTs or UCSs of group or individual psychological interventions using DBT, ACT, mindfulness-based therapies (e.g., MBSR, meditation, yoga, mindful eating, etc.), and assessed binge eating as an outcome variable. In the current review, binge eating was defined as eating an objectively large or excessive amount of food at one time and having a sense of loss of control. However, studies with participants in whom eating excessive amounts was not possible were included as well (e.g., bariatric surgery patients). Studies were excluded if they were book reviews, books, book chapters, published abstracts, conference proceedings, theses and dissertations, review articles, proof-of-concept papers, or treatment guidelines or manuals. Observational studies, case studies, single case experiments, and pharmacotherapy RCTs for binge eating were also excluded. Studies that examined binge eating only in the context of bulimia nervosa or anorexia nervosa were excluded. Studies with non-bariatric samples that only reported subjective bulimic episodes (SBE) or did not differentiate SBEs and objective bulimic episodes (OBE) were also excluded. Additionally, studies testing interventions that were within the ACT or DBT framework but did not include a mindfulness component (e.g., a DBT study that only introduced emotion regulation skills to patients), or did not include sufficient dosage of mindfulness practices (e.g., talking about mindful eating as a concept rather than actually doing mindfulness practices) were also excluded. Studies that only measured constructs related to binge eating (e.g., emotional eating, cravings, etc.), only assessed a history of binge eating rather than current binge eating, or only reported outcome data on subscales of measurements that did not directly assess binge eating (e.g., Eating Disorder Examination restraint subscale) were excluded as well. Finally, for the purpose of this review, studies that did not report sufficient data and authors did not respond to emails requesting the data were also excluded. Additionally, the current review also included the studies in the original review by Godfrey et al. (2015) so that this updated review can more comprehensively reflect the existing research efforts in this field.

### Search results, effect size, and quality of evidence assessment

**Fig. 1:**
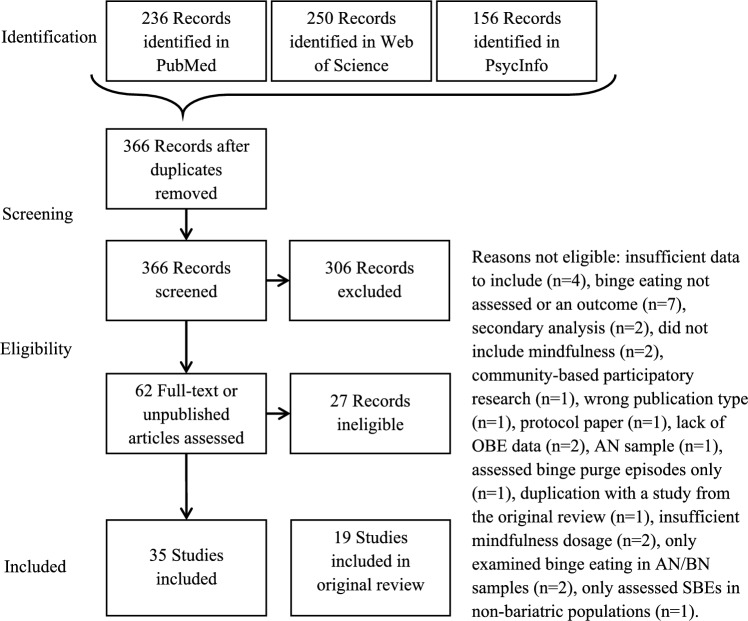
Flow of documents through the systematic review

Figure 1 presents the flow of documents through every stage of the review, including identification, screening, eligibility, and inclusion. The first four authors extracted data from these studies into a data collection table. Ten authors of the included studies were contacted via email to request additional study information. For the purpose of this review, effect sizes were manually calculated using means and standard deviations (SD) for all studies included. Within-group effect sizes were calculated using the difference between baseline to post-treatment and/or follow-up period. Between-group effect sizes were calculated by comparing the study groups at post-treatment and/or follow-up. All included studies were assessed for quality of evidence using the Effective Public Health Practice Quality Assessment Tool (EPHPP; Jackson et al., 2005), an assessment that has demonstrated a lower risk of bias than the Cochrane Collaboration Risk of Bias Tool (Armijo-Olivo et al., 2012).

### Statistical analysis

A meta-analysis was performed to examine the effectiveness of MBIs compared to control conditions. Separate analyses were conducted for the comparisons between MBIs with non-psychological intervention control groups (e.g., waitlist, no-intervention controls), and between MBIs with other psychological interventions (e.g., CBT). However, a meta-analysis was not conducted for within-group effect sizes, as recent recommendations have suggested that within-group pre-post effect sizes should be avoided for meta-analysis (Cuijpers et al., 2017). Random effects models were used for the analysis due to the methodological heterogeneity among studies, and overall *I*^*2*^ statistics were calculated to examine the degree of heterogeneity. Analyses were conducted in RStudio software version 2024.09.1 + 394 using the *metafor, dmetar, and meta* packages (Harrer et al., 2021). For studies that yielded multiple effect sizes, an aggregated effect size was calculated for each study at each time point (i.e., EOT and FU), as including multiple effect sizes from one study could bias the results (Borenstein et al., 2021).

## Results

### Study characteristics

Table 1 presents a detailed description of all the included studies. Of the 54 studies included in this review, 23 were RCTs, 24 were UCSs, and 7 were non-randomized cohort studies. Studies were published from 1999 to 2024. Twenty-six studies were conducted in the USA, 7 were from Canada, 5 were from Brazil, 4 were from the UK, 2 were from the Netherlands, 2 were from Portugal, 2 were from Sweden, and the remaining 6 from Australia, Chile, Denmark, Iran, Mexico, and New Zealand. Twenty-four studies recruited participants who endorsed binge eating and/or met diagnostic criteria for binge eating disorder (BED). Nineteen studies required participants to have a BMI in the overweight/obese range. Five studies recruited pre- and post-bariatric patients. The remaining studies did not have specific inclusion and exclusion criteria for the abovementioned features. Fifty-one studies used adult samples (min mean age = 22.4; max mean age = 58.46), and 3 studies used adolescent samples (min mean age = 15.4; max mean age = 16.2). The samples of the studies included in this review were mostly female (min % female = 13.3; max % female = 100) and had overweight or obesity (min mean BMI = 21.6; max mean BMI = 60.92).

**Table 1:** Information extracted from the studies on mindfulness-based interventions

Authors (year)	Study type and setting	Sample	Treatment and group size	Comparison(s) and group size	Key Findings
*RCTs*					
Afari et al. (2019)	RCT; USA	*N* = 88; BMI ≥ 25; 23.9% women; mean age 57.3 (*SD* = 9.9); “70.5% Caucasian, 19.3% African American, 13.6% Hispanic”	ACT Focus: address experiential avoidance in the context of eating behavior and factors that may lead to binge or other disinhibited eating *n* = 45; in-person, group sessions, 8 sessions, 2 h	BWL with some CBT techniques: *n* = 43	BES *M (SD)* Baseline: ACT 15.7 (9.2); BWL: 16.8 (9.0) Post-tx: ACT 13.6 (9.0); BWL: 10.6 (7.2) 3mfu: ACT 11.9 (8.6); BWL: 10.6 (7.4) 6mfu: ACT 11.9 (7.4); BWL: 10.0 (7.6) Effect sizes: ACT within d (baseline-post) = 0.12 Between post d = 0.37 Between 3mfu d = 0.16 Between 6mfu d = 0.25
Brennan et al. (2020)	RCT; Canada	*N* = 53; all met DSM-V BN or BED criteria; 100% women; 52.8% BMI normal; “71.7% Caucasian, 7.5% Asian Canadian, 5.7% Aboriginal/Metis, 3.8% East Indian, 1.9% Hispanic, 1.9% Chinese, 1.9% South East Asian, 5.7% Other”	Yoga Focus: addressing ED symptoms *n* = 26; in-person, group sessions, 8 sessions, 90 min	WL: *n* = 27; later offered access to active treatments	EDE-Q binge frequency *M (SD)* Baseline: Yoga 11.46 (7.54); WL 12.92 (7.79) Post-tx: Yoga 5.11 (5.45); WL 12.11 (10.22) 1mfu: Yoga 5.15 (7.79); WL 13.26 (11.96) Effect sizes: Yoga within d (baseline-post) = 0.97 Between post d = 0.85 EDE-Q binge days *M (SD)* Baseline: Yoga 11.63 (6.90); WL 11.70 (7.70) Post-tx: Yoga 4.58 (5.20); WL 10.60 (8.58) 1mfu: Yoga 5.63 (8.55); WL 11.50 (10.46) Effect sizes: Yoga within d (baseline-post) = 1.15 Between post d = 0.85
Cancian et al. (2019)	RCT; Brazil	*N* = 79; BMI ≥ 30; Tx group 100% women; Control group 88.23% women; Tx group mean age 39.50 (*SD* = 9.24); Control group mean age 40.11 (*SD* = 11.18)	DBT Focus: help managing problematic eating behaviors and increasing adaptive eating behaviors *n* = 30; in-person, group sessions, 10 sessions, twice a week, 2 h	WL: *n* = 30; later offered access to active treatment	BES *M (SD)* Baseline: DBT 26.2 (6.5); WL 22.8 (11) Post-tx: DBT 17.1 (7); WL 24.4 (10.7) Effect sizes: DBT within d (baseline-post) = 1.35 Between post d = 0.81
Carpenter et al. (2019)	RCT; USA	*N* = 75; BMI between 25 and 35; 92.0% women; mean age 47.3 (*SD* = 10.0); mean BMI 31.5 (*SD* = 2.3); “62% White non-Hispanic, 32% Black non-Hispanic, 4% Hispanic, 2% Asian”	MYW Focus: weight loss with mindfulness exercises *n* = 50; phone coaching and eLessons via email, 11 sessions, Weight Talk calls 1–4 30 min, the remaining calls 20 min	WT with CBT elements: *n* = 25	BES *M (SD)* Baseline: MYW 19.2 (6.8); WT 18.0 (7.5) 6mfu: MYW 11.5 (8.1); WT 15.9 (7.3) Effect sizes: MYW within d (baseline-6mfu) = 1.03 Between 6mfu d = − 0.57
Carter et al. (2020)	RCT; Canada	*N* = 71; all met DSM-V BED criteria; 92.96% women; mean age 40.70 (*SD* = 11.46); mean BMI 37.30 (*SD* = 9.40); “91.7% White/Caucasian, 8.3% Other”	DBT-GSH, DBT-USH Focus: addressing emotional eating DBT-GSH: *n* = 24; 12 weeks of self-help manual with 6 video-calling GSH sessions, 30 min DBT-USH: *n* = 24; 12 weeks of self-help manual	SE-USH: *n* = 23;	EDE binge frequency *M (SD)* Baseline: DBT-GSH 16.17 (10.17); DBT-USH 13.63 (10.93); SE-USH 21.74 (25.53) Post-tx: DBT-GSH 3.42 (6.69); DBT-USH 3.56 (5.22); SE-USH 4.90 (7.47) 3mfu: DBT-GSH 3.84 (6.97); DBT-USH 2.78 (5.96); SE-USH 5.15 (7.56) Effect sizes: DBT-GSH within d (baseline-post) = 1.48 DBT-GSH within d (baseline-3mfu) = 1.41 DBT-USH within d (baseline-post) = 1.18 DBT-USH within d (baseline-3mfu) = 1.23 DBT-GSH – SE-USH between post d = − 0.21 DBT-GSH – SE-USH between 3mfu d = − 0.18 DBT-USH – SE-USH between post d = − 0.21 DBT-USH – SE-USH between 3mfu d = − 0.35
Chen et al. (2017)	RCT; USA	*N* = 67; all met DSM-V BN or BED criteria; 100% women; Tx group mean age 38.2 (*SD* = 13.1); Control group mean age 37.8 (*SD* = 13.9)	DBT Focus: reducing binge eating n = 36; in-person, 6 months of weekly sessions; 2-h skill group, 1-h individual therapy	CBT: *n* = 31	# EDE OBE days *M (SD)* Baseline: DBT 12.31 (8.05); CBT 13.52 (6.32) Post-tx: DBT 4.31 (7.00); CBT 4.55 (7.06) 6mfu: DBT 4.97 (5.89); CBT 4.96 (7.38) 12mfu: DBT 4.61 (7.21); CBT 6.18 (6.59) Effect sizes: DBT within d (baseline-post) = 1.06 DBT within d (baseline-6mfu) = 1.04 DBT within d (baseline-12mfu) = 1.01 Between post d = − 0.03 Between 6mfu d = 0.001 Between 12mfu d = − 0.23 # EDE OBEs *M (SD)* Baseline: DBT 22.22 (27.30); CBT 21.65 (17.00) Post-tx: DBT 6.53 (16.42); CBT 5.00 (8.09) 6mfu: DBT 6.44 (9.21); CBT 7.78 (15.67) 12mfu: DBT 6.00 (12.09); CBT 6.18 (6.59) Effect sizes: DBT within d (baseline-post) = 0.70 DBT within d (baseline-6mfu) = 0.77 DBT within d (baseline-12mfu) = 0.77 Between post d = 0.12 Between 6mfu d = − 0.10 Between 12mfu d = − 0.02
Duarte et al. (2017)	RCT; Portugal	*N* = 20; all met DSM-V BED criteria; 100% women; Tx group mean age 37.73 (*SD* = 7.50); Control group mean age 35.78 (*SD* = 9.08); Tx group mean BMI 31.89 (*SD* = 6.25); Control group mean BMI 31.89 (*SD* = 6.25); “100% Caucasian”	CARE Focus: using mindfulness and compassion exercises to manage impulses to binge eat in the face of negative affectivity, shame, or self-critical thoughts *n* = 11; 1 2.5 h in-person lecture and 4 weeks of home practice delivered on a webpage	WL; *n* = 9	BES *M (SD)* Baseline: CARE 22.81 (7.41); WL 17.00 (5.77) Post-tx: CARE 12.00 (7.63); WL 15.66 (4.85) Effect sizes: CARE within d (baseline-post) = 1.44 Between post d = − 0.57 # EDE-Q binge episodes *M (SD)* Baseline: CARE 4.73 (1.62); WL 6.14 (2.04) Post-tx: CARE 1.27 (3.04); WL 5.14 (3.39) Effect sizes: CARE within d (baseline-post) = 1.42 Between post d = − 1.20
Katterman et al. (2014)	RCT; USA	*N* = 58; Interested in weight control; 100% women; mean age 22.4 (*SD* = 2.9); mean BMI 26.6 (*SD* = 2.2); “62% Caucasian, 11% African American, 11% Asian American/Pacific Islander, 7% Latino/Latina/Hispanic”	ACT and behavioral weight control Focus: healthy eating and exercise behaviors promoting long-term weight control *n* = 29; in-person, 8 session (first 4–5 weekly, rest monthly), 75 min	Control: *n* = 29; no treatment	OBE days *M* (*SD*) Baseline: ACT 0 (0); Control 0.5 (1.7) Mid-tx: ACT 0.2 (1.0); Control 0 (0) Post-tx: ACT 0.1 (0.2); Control 0.1 (0.6) Effect sizes: ACT within d (baseline-post) = − 0.71 Between post d = 0 # OBEs *M* (*SD*) Baseline: ACT 0 (0); Control 0.52 (1.7) Mid-tx: ACT 0.2 (1.0); Control 0 (0) Post-tx: ACT 0.1 (0.2); Control 0.1 (0.6) Effect sizes: ACT within d (baseline-post) = − 0.71 Between post d = 0
Kristeller et al. (2014)	RCT; USA	*N* = 140; 111 met DSM-IV or DSM-5 BED criteria; 88% women; mean age 46.6; mean BMI 40.3; “13% minority”	MB-EAT Focus: awareness of inappropriate eating patterns, tools and support to make sustainable changes *n* = 50; in-person, group tx; 9 weekly sessions then 3 monthly booster sessions for 12 sessions total. Sessions 1 and 6 were 2 h, rest were 1.5 h	WL: *n* = 42; later offered access to active treatments	EDE OBE days* M* (*SD*) Baseline: MB-EAT 14.8 (5.7); WL 14.0 (6.3) Post-tx: MB-EAT 4.8 (5.8); WL 12.8 (8.4) 4 or 6mfu: MB-EAT 3.8 (5.2); WL 11.4 (9.3) Effect sizes: MB-EAT within d (baseline-post) = 1.75 MB-EAT within d (baseline-6mfu) = 2.04 Between post d (MB-EAT – PECB) = − 0.07 Between 6mfu d (MB-EAT – PECB) = − 0.26 Between post d (MB-EAT – WL) = − 1.11 Between 6mfu d (MB-EAT – WL) = − 1.01 BES* M* (*SD*) Baseline: MB-EAT 29.0 (7.8); WL 28.1 (7.8) Post-tx: MB-EAT 15.2 (8.1); WL 25.9 (9.0) 4 or 6mfu: MB-EAT 13.5 (9.1); WL 25.1 (7.0) Effect sizes: MB-EAT within d (baseline-post) = 1.73 MB-EAT within d (baseline-6mfu) = 1.82 Between post d (MB-EAT – PECB) = − 0.31 Between 6mfu d (MB-EAT – PECB) = − 0.32 Between post d (MB-EAT – WL) = − 1.25 Between 6mfu d (MB-EAT – WL) = − 1.42
Lillis et al. (2011)	RCT; USA	*N* = 83; all completed at least 6 months of structured weight loss programs; 90% women; mean age 50.8 (*SD* = 11.3); mean BMI 33.0 (*SD* = 7.1)	ACT Focus: living a more fulfilling life consistent with chosen values *n* = 40; in-person, 1 workshop session of 6 h	WL: *n* = 43; completed the ACT workshop after the follow up	Weekly binge days *M* (*SD*) Baseline: ACT 1.8 (1.4); WL 1.8 (1.4) 3mfu: ACT 1.4 (1.5); WL 2.2 (1.9) Effect sizes: ACT within d (baseline-3mfu) = 0.28 Between 3mfu d = − 0.47
Masson et al. (2013)	RCT; Canada	*N* = 60; all with DSM-5 BED; 88% women; mean age 42.8 (*SD* = 10.5); mean BMI 38.0; “Tx group 93.1% Caucasian/White, 3.45% Middle Eastern, 3.45% Multiracial”	DBT Focus: reduce binge eating by teaching emotion regulation *n* = 30; guided self-help tx; One 45 min in-person session, 6 biweekly 20 min support phone calls over 13 weeks of guided self-help tx	WL:* n* = 30; given DBT tx after 13 weeks on WL	# EDE OBEs *M* (*SD*) Baseline: DBT 18.7 (13.2); WL 19.6 (11.9) Post-tx: DBT 6.0 (9.4); WL 14.4 (11.9) 6mfu: 9.5 (11.9) Effect sizes: DBT within d (baseline-post) = 1.11 DBT within d (baseline-6mfu) = 0.73 Between post d = − 0.87
Mercado et al. (2023)	RCT; UK	*N* = 45; BMI ≥ 25; 75.56% women; mean age 32 (median IQR = 13); mean BMI 34.3 (*SD* = 6.57); “55.6% White”	MT Focus: mindful eating and coping with cravings n = 16; 8 sessions; 10 min in-person training and home practice (with an APP component)	WL: n = 14; later offered access to treatment of their preference	# EDE-Q OBE days *M (SD)* Baseline: MT 6.5 (7.3); WL 7.8 (7.9) Post-tx: MT 4.4 (5.11); WL 2.63 (4.27) 1mfu: MT 3.4 (3.8); WL not reported Effect sizes: MT within d (baseline-post) = 0.33 MT within d (baseline-1mfu) = 0.53 Between post d = 0.38
Pepe et al. (2023)	RCT; Brazil	*N* = 138; 100% women; mean age 36.7; mean weight 89.43 kg; “57.1% Caucasian”	ME, ME and MER Focus: mindful eating exercises and healthy eating via the food pyramid adapted to the Brazilian population *n* = 43; 7 in-person group sessions, 90 min and at home practice (have smartphone messages and email components)	MER only: n = 49	BES *M(SD)* Baseline: ME 17.66 (10.22); ME + MER 15.79 (8.22); MER 15.13 (7.9) Post-tx: ME 10.05 (7.83); ME + MER 7.17 (6.26); MER 8.97 (7.31) Effect sizes: ME within d (baseline-post) = 0.84 ME + MER within d (baseline-post) = 1.18 Between post d (ME – MER) = 0.14 Between post d (ME + MER – MER) = 0.26
Potts et al. (2022)	RCT; USA	*N* = 55; BMI ≥ 27.5; 81.8% women; mean age 38.65 (*SD* = 12.40); mean BMI 37.01 (*SD* = 6.51); “100% White, 11.8% Hispanic/Latino”	ACT (phone coaching version and virtual coaching version) Focus: reduce harmful effects of weight stigma and develop more adaptive motivators for engaging in health behaviors Phone coaching (GSH-P): *n* = 17; 8 self-guided sessions with phone coaching Virtual coaching (GSH-E): *n* = 20; 8 self-guided sessions with email coaching	WL: *n* = 18	# EDE-Q OBEs *M (SD)* Baseline: GSH-P 4.12 (8.63); GSH-E 2.85 (3.82); WL 6.39 (9.57) Post-tx: GSH-P 0.25 (0.45); GSH-E 0.92 (1.93); WL 6.83 (9.02) Effect sizes: GSH-P within d (baseline-post) = 0.59 GSH-E within d (baseline-post) = 0.56 Between post d (GSH-P – WL) = 1.06 Between post d (GSH-E – WL) = 0.94
Rahmani et al. (2018)	RCT; Iran	*N* = 60; 100% women; mean age 29.66	DBT Focus: reducing binge eating, learning emotion regulation skills, weight loss *n* = 30; in-person, group sessions, 20 sessions, 2 h	WL: *n* = 30; later offered access to active treatment	BES *M (SD)* Baseline: DBT 23.80 (4.80); WL 22.53 (5.04) Post-tx: DBT 16.46 (2.19); WL 20.03 (2.68) Effect sizes: DBT within d (baseline-post) = 1.97 Between post d = − 1.46
Safer et al. (2010)	RCT; USA	*N* = 101 all with DSM-IV BED; 85% women, mean age 52.2 (*SD* = 10.6); mean BMI 36.4 (*SD* = 6.6); “76% Caucasian, 13% Latino, 5% Asian, 3% African American, 3% Unknown/Unreported”	DBT Focus: eliminate binge eating by improving emotion regulation *n* = 50; group tx; 20 weekly in-person sessions of 2 h each over 21 weeks (two weeks between sessions 19 and 20)	ACGT: *n* = 51 Supportive therapy	EDE OBE days* M* (*SD*) Baseline: DBT 15.3 (6.1) Post-tx: DBT 1.4 (2.8) 12mfu: DBT 2.6 (5.0) Effect sizes: DBT within d (baseline-post) = 2.93 DBT within d (baseline-12mfu) = 2.28 Between post d = − 0.78
Salvo et al. (2022)	RCT; Brazil	*N* = 284; 100% women; mean age 40.4 (*SD* = 10.7); mean BMI 32.7 (*SD* = 3.8)	MB-EAT Focus: developing a greater sense of self-awareness and self-acceptance regarding eating and weight *n* = 95; in-person, group sessions, 10 sessions, session duration not reported MBHP Focus: practicing mindfulness activities and increasing physical activity *n* = 93; 8 weeks of self-practice with clinicians’ suggestions for activities	TAU: *n* = 96; BWL with different levels of services based on comorbidities and BMI	BES *M (SD)* Baseline: ME-EAT 15.63 (8.94); MBHP 14.85 (8.82); TAU 13.79 (8.58) Post-tx: MB-EAT 8.74 (6.39); MBHP 12 (8.47); TAU 11.7 (8.55) 3mfu: MB-EAT 9 (8.09); MBHP 9.8 (6.59); TAU 12.13 (9.08) Effect sizes: MB-EAT within d (baseline-post) = 0.89 MB-EAT within d (baseline-6mfu) = 0.78 MBHP within d (baseline-post) = 0.33 MBHP within d (baseline-6mfu) = 0.65 Between post d (MB-EAT – TAU) = 0.39 Between 6mfu d (MB-EAT – TAU) = 0.36 Between post d (MBHP – TAU) = 0.03 Between 6mfu d (MBHP – TAU) = 0.29
Smith et al. (2018)	RCT; Mexico	*N* = 36; BMI ≥ 30; 100% women; mean age 58.46 (*SD* = 4.87); mean weight 95.58 kg (*SD* = 17.22)	MEAL Focus: cultivating an increased awareness of eating behavior and providing participants with greater control over eating *n* = 18; in-person, group sessions, 6 sessions, 2 h	Control: *n* = 18; MEAL intervention without a mindfulness component	BES *M (SD)* Baseline: MEAL 16.94 (8.24); Control 12.66 (7.42) Post-tx: MEAL 8.37 (4.59); Control 8.76 (5.94) 4mfu: MEAL 9.26 (4.99); Control: 7.47 (5.34) 9mfu: MEAL 10.57 (7.23); Control 7.72 (5.81) Effect sizes: MEAL within d (baseline-post) = 1.28 MEAL within d (baseline-4mfu) = 1.13 MEAL within d (baseline-9mfu) = 0.82 Between post d = − 0.07 Between 4mfu d = 0.35 Between 9mfu d = 0.5
Strandskov et al. (2017)	RCT; Sweden	*N* = 92; all met DSM-V BN or EDNOS criteria; 96.7% women; mean age 29.14 (*SD* = 9.69); mean BMI 25.45 (*SD* = 5.91)	ACT-influenced CBT Focus: Internet based; teaching skills for willingness, mindfulness in eating, and remaining detached to distressing thoughts concerning the body and eating *n* = 43; Internet-based self-guided sessions, 8 sessions, duration not reported	WL: *n* = 46	#EDE-Q OBEs *M(SD)* Baseline: ACT 10.85 (10.53); WL 9.00 (8.93) Effect sizes: ACT within d (baseline-post) = 0.28 Between post d = 0.07
Tapper et al. (2009)	RCT; UK	*N* = 62; all actively attempting to lose weight; 100% women, mean age 41 (*SD* = 13), mean BMI 31.7 (*SD* = 6.1)	ACT Focus: enhance motivation, reduce associations between food- and exercise-related thoughts and behaviors, build tolerance of negative feelings *n* = 31; 4 2-h in-person workshop sessions total: 3 weeks consecutive then 1 follow up session 3 months later	TAU: *n* = 31; continue weight loss attempts, given a chance to attend a 1-day weight loss workshop at end of study	Shortened BES* M* (*SD*) Baseline: ACT 7.9 (3.9); TAU 9.1 (3.5) 4mfu: ACT 6.7 (3.6); TAU 9.4 (3.8) 6mfu: ACT 5.4 (3.5); TAU 10.1 (4.4) Effect sizes: ACT within d (baseline-4mfu) = 0.32 ACT within d (baseline-6mfu) = 0.67 Between 4mfu d = − 0.73 Between 6mfu d = − 1.18
Telch et al. (2001)	RCT; USA	*N* = 44; all with DSM-IV BED; 100% women, mean age 50 (*SD* = 9.1); mean BMI 36.4 (*SD* = 6.6); “94% Caucasian”	DBT Focus: eliminate binge eating by improving emotion regulation *n* = 18; in-person, group tx; 20 weekly in-person sessions of 2 h each	WL: *n* = 16; given a chance to complete DBT intervention at post-tx	EDE OBE days *M* (*SD*) Baseline: DBT 10.5 (9.0); WL 14.0 (5.0) Post-tx: DBT 0 (0); WL 8.5 (10) Effect sizes: DBT within d (baseline-post) = 1.65 Between post d = − 1.20 # EDE OBEs *M* (*SD*) Baseline: DBT 11.5 (10.8); WL 14.5 (7.5) Post-tx: DBT 0 (0); WL 10 (14) Effect sizes: DBT within d (baseline-post) = 1.51 Between post d (baseline-post) = − 1.01 BES *M* (*SD*) Baseline: 28.8 (6.1); WL 31.8 (6.0) Post-tx: DBT 15.7 (9.4); WL 28.2 (8.3) Effect sizes: DBT within d (baseline-post) = 1.65 Between post d = − 1.41
Ugarte Perez et al. (2023)	RCT; Chile	*N* = 98; BMI ≥ 24.9; 76% women; mean age 34.8 (*SD* = 10.2); mean BMI 31.71 (*SD* = 5.06)	MB-EAT Focus: increasing mindful awareness of eating-related experiences and reducing emotionally and contextually triggered eating *n* = *20*; video-conferencing group sessions, 8 sessions, 2 h	Control: *n* = *21*; BWL	BES *M (SD)* Baseline: MB-EAT 20 (7.30); BWL 19.74 (10.52) Post-tx: MB-EAT 14.14 (7.60); BWL 13.21 (7.16) Effect sizes: MB-EAT within d (baseline-post) = 0.79 Between post d = 0.13
Weineland et al., (2012a, 2012b)	RCT; Sweden	*N* = 39; all post-bariatric surgery patients; 90% female; mean age 43.1; mean BMI preoperative 37.1, mean BMI at study baseline 27.2	ACT Focus: increase conscious valued life quality *n* = 19; 2 in-person sessions (1.5 h) at start and end of tx, 6-week self-help tx via internet modules, weekly 30 min support phone session	TAU: *n* = 20; dietary guidelines, follow up and in person telephone sessions as needed, conducted by bariatric team (surgeon, nurse, dietician)	DEBS *M* (*SD*) Baseline: ACT 4.1 (4.1); TAU 5.2 (5.2) Post-tx: ACT 1.6 (2.4); TAU 5.54 (5.9) Effect sizes: ACT within d (baseline-post) = 0.74 Between post d = − 0.87
*UCSs*					
Adams et al. (2021)	UCS; UK	*N* = 84; 94% women; mean age 36.67 (*SD* = 11.82); mean BMI 38.90 (*SE* = 2.44)	DBT Focus: 10-week DBT-BED skills training group *n* = 84; in-person, group sessions, condensed 10 sessions, 2 h		BES *M (SE)* Baseline: 35.0 (0.72) Post-tx: 15.8 (2.11) 1mfu: 13.4 (2.94) Effect sizes: DBT within d (baseline-post) = 1.35 DBT within d (baseline-fu) = 0.91
Baer et al. (2005a)	UCS; USA	*N* = 10; 60% met DSM-IV BED criteria 40% met criteria except for frequency of binges (only 3–5 in past month); 20% previous BN symptoms; 100% women; age range 23–65; BMI range 22–40; “90% White, 10% biracial”	MBCT Focus: mindfulness and cognitive-based skills to reduce binge eating *n* = 6 completers; group tx; in-person, 10 weekly sessions, 2 h in length		# OBEs* M* Baseline: 15.7 Post-tx: 4.0 BES *M* Baseline: 25.8 Post-tx: 18.4 *Insufficient data to calculate effect sizes*
Blood et al. (2020)	UCS; USA	*N* = 56; all met DSM-V BED criteria; 89% women; mean age 37.96 (*SD* = 11.57); mean BMI 42.2 (*SD* = 8.73)	DBT Focus: reducing binge eating *n* = 56; in-person, group sessions, 20 sessions, 2 h		BES *M (SE)* Baseline: 34.3 (0.85) Post-tx: 11.7 (6.48) 1mfu: 13.5 (5.70) Effect size: DBT within d (baseline-post) = 0.66 # EDE-Q OBEs *M (SE)* Baseline: 18.6 (1.72) Post-tx: 1.34 (5.94) 1mfu: 6.29 (34.3) Effect size: DBT within d (baseline-post) = 0.53
Boucher et al. (2016)	UCS; New Zealand	*N* = 40; BMI ≥ 26.5; 100% women; mean age 44.8 (*SD* = 3.06); mean BMI 32.9 (*SD* = 6.01); “81.1% New Zealand European, 10.8% Māori, 2.7% Pacific”	ACT Focus: skills related to intuitive eating *n* = 40; web-based self-guided sessions, 12 sessions, 15–20 min		# EDE-S OBE *M (SD)* Baseline: 7.1 (5.8) Post-tx: 4.7 (6.4) 3mfu: 5.3 (7.2) Effect sizes: ACT within d (baseline-post) = 0.39 ACT within d (baseline-3mfu) = 0.28
Braden et al. (2022)	UCS; USA	*N* = 39; BMI ≥ 25; 97.4% women; mean age 49.21 (*SD* = 10.91); mean BMI 35.77 (*SD* = 6.84); “91.7% Caucasian, non-Hispanic”	DBT Focus: behavioral weight loss and DBT techniques *n* = 39; in-person, group sessions, 16 sessions, 2 h		BES *M (SD)* Baseline: 22.67 (6.67) Post-tx: 12.69 (7.84) 6mfu: 13.62 (7.86) Effect size: DBT within d (baseline-post) = 1.37
Chen et al. (2008)	UCS: USA	*N* = 8; all had BPD; *n* = 5 had BED; *n* = 3 had BN; all women; median age 31; mean BMI 35.8 (*SD* = 6.4); “87.5% Caucasian, 12.5% Korean-American”	DBT Focus: standard DBT modified to address binge eating *n* = 8; in-person, weekly DBT (skills group, individual therapy, 24 h telephone access) for 6 months		# EDE OBEs *M* (*SD*) Baseline: 16.0 (10.4) Post-tx: 5.3 (10.1) 6mfu: 5.8 (9.9) Effect sizes: DBT within d (baseline-post) = 1.04 DBT within d (baseline-6mfu) = 1.00
Courbasson et al. (2011)	UCS; Canada	*N* = 38; 79% women; all met criteria for SUD; mean age 42 (*SD* = 11.0)	MACBT Focus: build skills in mindfulness including emotion regulation and mindful eating, psychoeducation, balanced physical activity, focusing on individual strengths *n* = 38; in-person, 16 weekly 2-h group sessions		# EDE-Q OBEs *M* (*SD*) Baseline: 19.1 (4.5) Post-tx: 8.1 (2.6) Effect size: MACBT within d (baseline-post) = 2.99
Cuneo et al. (2018)	UCS; USA	*N* = 85; 86.7% men; mean age 57.9 (*SD* = 8.5); mean BMI 38 (*SD* = 6)	ACT Focus: increasing awareness of the relationship between eating and stress and other emotions to learn healthier ways to cope with stress *n* = 85; in-person, group sessions, 6–8 sessions, 90 min		BES *M (SD)* Baseline: 20.87 (8.21) Post-tx: 16.03 (8.6) Effect size: d (baseline-post) = 0.58
Dalen et al. (2010)	UCS; USA	*N* = 10; 70% women; mean age 44 (*SD* = 8.7); mean BMI 36.9 (*SD* = 6.3); “60% Caucasian, 20% Hispanic”	MEAL Focus: cultivate awareness of behaviors and reduce automatic eating to reduce binge-type eating and improve psychological functioning *n* = 10; group tx; 6 weekly sessions lasting 2 h each		BES *M* (*SD*) Baseline: 16.2 (5.4) Post-tx: 9.2 (5.1) 3mfu: 7.2 (2.3) Effect sizes: MEAL within d (baseline-post) = 1.33 MEAL within d (baseline-3mfu) = 2.17
de Souza et al. (2019)	UCS; Brazil	*N* = 121; all met DSM-V BED criteria; 95% women; mean age 38.49 (*SD* = 10.54); mean BMI 38.56	DBT Focus: teaching emotional regulation skills to help individuals manage their problematic eating behaviors and increase adaptive eating behaviors *n* = 121; in-person, group sessions, 20 sessions, 2 h		BES *M (CI)* Baseline: 24.33 (21.94–26.52) Post-tx: 16.44 (12.85–20.03) 3mfu: 16.06 (11.96–20.16) 8mfu: 18.62 (13.33–23.91) Effect size (author-reported): R^2^ = 0.15
Fischer & Peterson, (2015)	UCS; USA	*N* = 7; all were classified as overweight or obese; mean age 16.20 (1.03); “100% Caucasian”	DBT Focus: addressing binge eating, purging, suicidal behavior and non-suicidal self-injury *n* = 7; in-person, 24 individual therapy and 24 group therapy		# EDE binge episodes *M (SD)* Baseline: 9.29 (6.90) Post-tx: 4.57 (11.19) 6mfu: 0.50 (0.55) Effect sizes: DBT within d (baseline-post) = 0.47 DBT within d (baseline-6mfu) = 1.39
Kamody et al. (2019)	UCS; USA	*N* = 15; 73.33% girls; mean age 15.40 (*SD* = 1.30); mean BMI z-scores 2.36 (*SD* = 0.62); “100% African American/Black”	DBT Focus: reducing binge eating *n* = 15; in-person, group sessions, 8 active treatment sessions, 1 h		# EDE-Q OBEs *M (SD; authors did not report SD for post-tx and 3mfu)* Baseline: 3.67 (4.95) Post-tx: 2.13 3mfu: 1.36 I*nsufficient data to calculate effect sizes*
Karam et al. (2022)	UCS; USA	*N* = 454; 91.63% women; mean age 26.15 (*SD* = 9.14); mean BMI 21.60 (*SD* = 5.34); 28.4% AN-R, 14.8% AN-BP, 30.4% BN, 2.1% BED, 19.2% OSFED, 4.2% ARFID; “82.3% White, 4.3% Asian, 1.3% Black, 1.3% Native American/Alaskan Native, 0.9% Native Hawaiian/Pacific Islander, 9.9% Other, 16.7% Hispanic”	DBT Focus: addressing ED symptoms *n* = 454; in-person, PHP with group and individual sessions		# EDE-Q OBEs *M (SD)* Baseline: 8.01 (13.46) Mid-tx: 2.36 (5.44) Post-tx: 1.44 (3.55) Effect size: DBT within d (baseline-post) = 0.67
Klein et al. (2012)	UCS; USA	*N* = 10; all reported binge eating; 80% met full or partial criteria for BED; 20% BN; 100% women; mean age 39.6 (*SD* = 5.6); “100% White”	DBT Focus: group DBT for binge eating *n* = 5; treatment completers; in-person, group tx; 16 weekly sessions over 18 weeks (two week break at midway point) each 2–2.5 h, coaching calls between sessions		Self-reported weekly binges *M* (*SD*) Baseline: 3.4 (1.8) Post-tx: 0.5 (0.6) Effect size: DBT within d (baseline-post) = 2.16
Kristeller and Hallett (1999)	UCS; USA	*N* = 18; all met DSM-IV BED criteria; 100% women; mean age 46.5 (*SD* = 10.5); mean BMI 40.3; “94.4% White”	MB-EAT Focus: use of general mindfulness meditation, eating meditation, and mini-meditation *n* = 18; in-person, group tx; 7 sessions over 6 weeks		Self-reported weekly binges *M* (*SD*) Baseline: 4.0 (1.4) Post-tx: 1.6 (1.5) Effect size: MB-EAT within d (baseline-post) = 1.65 BES *M* (*SD*) Baseline: 31.7 (7. 7) Post-tx: 15.1 (8.1) Effect size: MB-EAT within d (baseline-post) = 2.10
Leahey et al. (2008)	UCS; USA	*N* = 7; all post-bariatric surgery patients; 85% women; mean age 54; mean BMI 40.8 (*SD* = 5.4); “85.7% Caucasian”	CB mindfulness-based intervention Focus: decrease binge eating and emotional eating; enhance well-being and postsurgical adjustment *n* = 7; in-person, group tx; 10 weekly sessions lasting 75 min each		Loss of control *M* (*SD*) Baseline: 9.1 (7.7) Post-tx: 0.4 (0.7) Effect size: MBCT within d (baseline-post) = 1.59 Guilt after eating Baseline: 2.3 (1.6) Post-tx: 0.6 (0.5) Effect size: MBCT within d (baseline = post) = 1.43
Mensinger, (2022)	UCS; USA	*N* = 70; 97.1% women; mean age 45.5 (*SD* = 10.9); mean BMI 33.7 (*SD* = 8.0); “87.1% White, 5.7% Hispanic/Latinx, 4.3% Mixed race, 1.4% Other”	NMW Focus: healing one’s relationship to food and the body *n* = 70; 6 self-guided e-course modules		# EDE-Q OBEs *M (SE)* Baseline: 0.50 (0.06) Post-tx: 0.25 (0.07) Effect size (author-reported): NMW within d (baseline-post) = medium
Minari et al. (2024)	UCS; Brazil	*N* = 82; all met DSM-V BED criteria; all classified as obese; 57.3% women; mean age 47.5 (*SD* = 4.8); mean BMI 37.3 (*SD* = 3.9); “19.5% White, 22% Black, 17.1% Brown, 14.6% Indigenous, 26.8% Yellow”	ME Focus: encourage patients to consume fewer ultra-processed foods and use ME during daily meals n = 82; in-person, 8 individual sessions		BES *M (SD)* Baseline: 33.2 (3.3) 8th week: 31 (3.3) Effect size: ME within d (baseline-8th week) = 0.67 # Self-reported binge episodes *M (SD)* Baseline: 8 (2.3) 8th week: 3 (1.4) Effect size: ME within d (baseline-8th week) = 2.63
Moffat et al. (2019)	UCS; UK	*N* = 166; Interested in weight loss; 80% women; mean age 47.5 (*SD* = 13.65); mean BMI 48.2 (*SD* = 9.36); “89.2% White British/Irish, 1.2% Other”	ACT Focus: develop mindfulness strategies to target experiential avoidance and focus on behaviors that move patients towards valued outcomes *n* = 166; in-person, group sessions, 12 sessions, 2 h		BES *M (SD)* Baseline: 20.54 (8.45) 6mfu: 13.9 (7.74) Effect size: ACT within d (baseline-6mfu) = 0.82
Mushquash and McMahan (2015)	UCS; Canada	*N* = 11; all met DSM-V BED criteria; bariatric surgery candidates; 90.9% women; mean age 44.56 (*SD* = 16.31); mean BMI 60.92 (*SD* = 8.84); “81.8% Caucasian”	DBT Focus: address factors that are relevant to the occurrence and maintenance of binge eating including tendencies to eat mindlessly and turning to food to cope with unpleasant emotions, crises, and problems in relationships *n* = 11; in-person, group sessions, 10 sessions, 2 h		BES *M (SD)* Baseline: 28.72 (4.88) Post-tx: 22.06 (7.79) Effect size: DBT within d (baseline-post) = 1.02
Smith et al. (2006)	UCS; USA	*N* = 25; community sample signing up for a fee-based stress reduction course; 80% women; mean age 47.8 (*SD* = 13.1); mean BMI 27.9 (*SD* = 7.4)	MBSR Focus: increase mindfulness with a focus on eating *n* = 25; in-person, group course; 8 weekly sessions each lasting 3 h with a one-day full retreat		BES *M* (*SD*) Baseline: 10.1 (9.6) Post-tx: 7.1 (7.1) Effect size: MBSR within d (baseline-post) = 0.36
Telch et al. (2000)	UCS; USA	*N* = 11; all met DSM-IV criteria for BED; 100% women; mean age 45 (*SD* = 11.7); “90.9% White non-Hispanic, 9.1% Pacific Islander”	DBT Focus: eliminate binge eating by improving emotion regulation *n* = 18; in-person, group tx; 20 weekly in-person sessions of 2 h each		EDE OBE days *M* (*SD*) Baseline: 11.8 (6.0) Post-tx: 1.8 (4.7) Effect size: DBT within d (baseline-post) = 1.86 # EDE OBEs *M* (*SD*) Baseline: 15.2 (12.3) Post-tx: 3.2 (7.6) Effect size: DBT within d (baseline-post) = 1.17 BES *M* (*SD*) Baseline: 32.4 (8.5) Post-tx: 17.2 (9.6) Effect size: DBT within d (baseline-post) = 1.68
Wnuk et al., (2018)	UCS; Canada	*N* = 22; bariatric surgery patients; 100% women; mean age 55.41 (*SD* = 9.44); mean BMI 32.82 (*SD* = 5.31); “95% Caucasian”	MB-EAT Focus: cultivating the ability to integrate patients’ own food preferences, physical, and emotional needs with knowledge about healthy eating when making decisions about eating *n* = 22; in-person, group sessions, 8 sessions, 2 h		BES *M (SD)* Baseline: 17.65 (11.44) Post-tx: 14.76 (10.91) Effect sizes: MB-EAT within d (baseline-post) = 0.26 MB-EAT within d (baseline-4mfu) = 0.19
Woolhouse et al. (2012)	UCS; Australia	*N* = 30; 50% had symptoms of DSM-IV BED; 31% had BN symptoms, 19% had sub-clinical symptoms; 100% women; mean age 32.2 (*SD* = 7.9)	Mindful MEG Focus: better understand and control eating behavior *n* = 30; in-person, group tx; 10 weekly sessions of 3 h duration		MAEDS binge eating *M* (*SD*) Baseline: 4.5 (0.9) Post-tx: 2.9 (1.2) 3mfu: 2.9 (1.3) Effect sizes: Mindful MEG within d (baseline-post) = 1.51 Mindful MEG within d (baseline-3mfu) = 1.43
*CAs*					
Barnes and Kristeller (2016)	CA; USA	*N* = 40; 65% women; mean age 16.2 (*SD* = 1.2); mean BMI = 32.4 (*SD* = 9); “2.5% Caucasian, 87.5% African American”	MB-EAT Focus: managing eating awareness, reducing stress and flexibly improving dietary and exercise patterns *n* = 18; in-person, group sessions, 12 sessions, 45 min	HE *n* = 22	BES *M (SD)* Baseline: MB-EAT 9.9 (7.2); HE 15.2 (10.2) Post-tx: MB-EAT 10.9 (7.3); HE 10.6 (0.9) Effect sizes: MB-EAT within d (baseline-post) = − 0,14 Between post d = 0.03
Corazon et al. (2018)	CA; Denmark	*N* = 15; all met DSM-V BED criteria; Tx group 100% women; Control group 85.71% women; Tx group mean age 47; Control group mean age 41	NBT Focus: guided body and mindful awareness exercises in the natural environment *n* = 8; in-person, group sessions, 12 sessions, 3 h	Control: *n* = 7; support group meetings	EDE binge frequency *M (SD)* Baseline: NBT 21.5 (29.56); Control 13.7 (10.31) Post-tx: NBT 3.5 (4.38); Control 10.9 (8.97) Effect sizes: NBT within d (baseline-post) = 0.85 Between post d = − 1.05
Delparte et al. (2019)	CA; Canada	*N* = 95; Bariatric surgical candidates; 80% women; mean age 44.4 (*SD* = 10.1); mean BMI 50.7 (*SD* = 9.1); “86.3% Caucasian, 2.1% African American, 11.6% First Nations or Metis”	DBT Focus: skills training tailored to the special needs of bariatric surgical candidate population (i.e., eating pathology) n = 50; video-conferencing group sessions, 8 sessions, 1 h 45 min	TAU: n = 45; Dietary counseling and eudcation	BES *M (SD) (Authors only reported baseline data)* Baseline: DBT 18.88 (8.87); TAU: 16.44 (7.71) Effect size (author-reported): Between post d = 0.006
Lammers et al. (2020)	CA; Netherlands	*N* = 74; all met DSM-V BED criteria; BMI ≥ 30; 89.2% women; mean age 37.3 (*SD* = 11.8); mean BMI 39.9 (*SD* = 5.6)	DBT-BED Focus: help patients regulate emotions in an adaptive way *n* = 41; in-person, group sessions, 20 sessions, 2 h	TAU: *n* = 33; CBT	# EDE-Q OBEs *M (SD)* Baseline: DBT-BED 7.51 (8.72); TAU 8.27 (9.65) Post-tx: DBT-BED 1.64 (3.77); TAU 0.74 (1.68) 6mfu: DBT-BED 2.75 (5.58); TAU 1.85 (5.11) Effect sizes: DBT within d (baseline-post) = 0.87 DBT within d (baseline-6mfu) = 0.65 Between post d = 0.31 Between post d = 0.17
Lammers et al. (2022)	CA; Netherlands	*N* = 175; all met DSM-V BED or subthreshold BED criteria; 89.1% women; mean age 34.9 (*SD* = 10.9); mean BMI 42.3 (*SD* = 7.6)	DBT-BED Focus: help patients replace binge eating, as a way of coping with negative affect, by adequate emotion regulation skills *n* = 42; in-person, group sessions, 20 sessions, 2 h	TAU: *n* = 133; CBT	# EDE-Q OBEs *M (SE)* Baseline: DBT-BED 5.18 (1.09); TAU 5.48 (0.61) Post-tx: DBT-BED 1.32 (0.03); TAU 0.90 (0.11) 6mfu: DBT-BED 1.31 (0.30); TAU 1.15 (0.14) Effect sizes (author-reported): Between post d = 0.19 Between 6mfu d = 0.09
Pinto-Gouveia et al. (2017)	CA; Portugal	*N* = 59; all met DSM-V BED criteria; 100% women	BEfree Focus: addressing binge eating and weight loss *n* = 19; in-person, group sessions, 12 sessions, 2.5 h	WL: *n* = 17	BES *M (SD)* Baseline: BEfree 29.94 (10.98); WL 28.65 (7.85) Post-tx: BEfree 12.83 (6.65); WL 26.35 (8.93) Effect sizes: BEfree within d (baseline-post) = 1.88 Between post d = 1.72
Smith et al. (2008)	CA; USA	*N* = 50; community sample choosing one of two fee-based stress reduction courses; 80% women; mean age 44.9 (*SD* = 13.7)	MBSR Focus: increase mindfulness with a focus on eating *n* = 36; in-person, group course; 8 weekly sessions each lasting 3 h with a one-day full retreat on week 6	CBSR: *n* = 14; group course; 8 weekly sessions each lasting 3 h	BES *M* (*SD*) Baseline: MBSR 1.8 (0.6); CBSR 1.5 (0.5) Post-tx: MBSR 1.6 (0.4); CBSR 1.4 (0. 5) Effect sizes: MBSR within d (baseline-post) = 0.39 Between post d = 0.44

Effect sizes are Cohen’s d calculated using Ms and SDs. Racial and ethnic data were self-reported by study participants in each included study. RCT is randomized controlled trial. UCS is uncontrolled cohort study. CA is cohort analytic study (two non-randomized groups assessed pre- and post-tx). SE is standard error. CI is confidence interval. BMI is body mass index. ACT is Acceptance and Commitment Therapy. BWL is behavioral weight loss. PHP is partial hospitalization program. MEAL is mindful eating and living. CBT is cognitive-behavioral therapy. MBSR is mindfulness-based stress reduction. CBSR is cognitive-based stress reduction. MB-EAT is mindfulness-based eating awareness training. MBCT is mindfulness-based cognitive therapy. DBT is Dialectical Behavior Therapy. HE is health education. MYW is Mind Your Weight. WT is Weight Talk. GSH is guided self-help. USH is unguided self-help. SE-USH is self-esteem unguided self-help. NBT is nature-based therapy. CARE is compassionate attention and regulation of eating behavior. NMW is No More Weighting. MT is mindfulness training. ME is mindful eating. MER is moderate energy restriction. MBHP is Mindfulness-Based Health Promotion. BED is binge eating disorder. BN is bulimia nervosa. AN-R is anorexia nervosa-restrictive subtype. AN-BP is anorexia-binge/purge subtype. OSFED is other specified feeding and eating disorder. ARFID is avoidant/restrictive food intake disorder. MEG is the moderate eating program. PECB is psycho-educational cognitive behavioral therapy. WL is waitlist. TAU is treatment as usual. Tx is treatment. Mfu is month follow up. TAU is treatment as usual. OBE is objective bulimic episode. EDE is eating disorder examination. EDE-Q is eating disorder examination-questionnaires. EDE-S is eating disorder examination-screening version. BES is the binge eating scale. EDI-3 SC is the eating disorders inventory-3 symptom checklist. MAEDS is the multifactorial assessment of eating disorders scale. DEBS is disorder eating after bariatric surgery self-report questionnaire. SUD is substance use disorder. MACBT is Mindfulness-action based Cognitive Behavioral Therapy. BPD is borderline personality disorder.

### Quality of evidence

Table 2 presents the component and global rates of quality assessment for each study. The 19 studies from the original review received the same ratings. Twelve of the 35 newly included studies received weak global quality ratings, mostly due to weak scores on the selection bias (e.g., low percentage of eligible participants deciding to enroll in the study), rating of confounders (e.g., no mentioning of including covariates in the analyses to control for confounders), and rating of withdrawal (e.g., no mention of reasons why participants withdrew and a low percentage of participants completing assessments). Seventeen studies received moderate global quality ratings, and the remaining 6 studies received strong ratings of global quality. Taken together, the overall rating of quality for the studies reviewed was moderate (mean global rating = 2.17). Three studies (Afari et al., 2019; Cuneo et al., 2018; Karam et al., 2022) were conducted by or in collaboration with researchers at the University of California San Diego and 4 studies (Adams et al., 2021; Blood et al., 2020; Mercado et al., 2023; Moffat et al., 2019) were conducted in collaboration with the National Health Service (NHS). Five studies were conducted by researchers in Brazil (de Souza et al., 2019; Minari et al., 2024; Pepe et al., 2023; Salvo et al., 2022), 2 studies were conducted by or in collaboration with University of Regina (Brennan et al., 2020; Delparte et al., 2019), and 2 studies were conducted by the same research group based in the Netherlands (Lammers et al., 2020, 2022). Compared to the original review by Godfrey & colleagues (2015), the quality of evidence has strengthened in that 6 studies received strong global quality scores compared to none. Furthermore, roughly 34% of the studies reviewed received weak ratings in this update compared to 42%. However, just as in the original review, the overall rating of quality for the studies was moderate.

**Table 2:** Component and global ratings of quality of evidence using EPHPP

Authors (Year)	Selection bias	Study design	Confounders	Blinding	Data collection method	Withdrawals and dropouts	Global rating
Adams et al., (2021)	2	1	NA	NA	1	2	1
Afari et al., (2019)	2	1	3	1	1	1	2
Barnes and Kristeller (2016)	3	1	3	2	1	3	3
Blood et al., (2020)	2	1	NA	NA	1	2	1
Boucher et al., (2016)	3	2	NA	NA	1	2	2
Braden et al., (2022)	3	2	NA	NA	1	1	2
Brennan et al., (2020)	3	1	1	2	1	2	2
Cancian et al., (2019)	3	1	2	2	1	3	3
Carpenter et al., (2019)	3	1	1	2	1	1	2
Carter et al., (2020)	3	1	3	2	1	1	3
Chen et al., (2017)	3	1	1	2	1	1	2
Corazon et al., (2018)	3	2	3	2	1	2	3
Cuneo et al., (2018)	3	2	NA	NA	1	1	2
de Souza et al., (2019)	3	2	NA	NA	1	3	3
Delparte et al., (2019)	2	1	1	NA	1	1	1
Duarte et al., (2017)	3	2	1	2	1	2	2
Fischer and Peterson, (2015)	2	2	NA	NA	1	1	1
Kamody et al., (2019)	3	2	NA	NA	1	2	2
Karam et al., (2022)	2	2	NA	NA	1	3	2
Lammers et al., (2022)	3	2	1	2	1	3	3
Lammers et al.,(2020)	3	2	1	2	1	2	2
Mensinger (2022)	3	2	NA	NA	1	3	3
Mercado et al., (2023)	3	1	3	3	1	2	3
Minari et al., (2024)	3	2	NA	NA	1	1	2
Moffat et al., (2019)	3	2	NA	NA	1	3	3
Mushquash and McMahan, (2015)	3	2	NA	NA	1	1	2
Pepe et al., (2023)	3	1	3	2	1	3	3
Pinto-Gouveia et al., (2017)	3	1	N/A	2	1	2	2
Potts et al., (2022)	3	1	1	2	1	2	2
Rahmani et al., (2018)	2	1	1	2	1	1	1
Salvo et al., (2022)	1	2	1	2	1	3	2
Smith et al., (2018)	3	1	3	2	1	1	3
Strandskov et al., (2017)	3	1	1	2	1	2	1
Ugarte Perez et al., (2023)	3	1	1	2	1	3	3
Wnuk et al., (2018)	1	2	N/A	2	1	3	2
Studies included in the original review (Godfrey et al., 2015)							
Baer et al., (2005b)	3	2	N/A	2	1	2	2
Chen et al., (2008)	3	2	N/A	2	1	2	2
Courbasson et al., (2011)	3	2	N/A	2	1	2	2
Dalen et al. (2010)	3	2	N/A	2	1	1	2
Katterman et al., (2014)	3	1	1	2	1	2	2
Klein et al., (2012)	3	2	N/A	2	3	3	3
Kristeller and Hallett (1999)	3	2	N/A	2	1	1	2
Kristeller et al., (2014)	3	1	3	2	1	2	3
Leahey et al., (2008)	3	2	N/A	2	3	3	3
Lillis et al., (2011)	3	1	1	2	3	1	3
Masson et al., (2013)	3	1	3	1	1	2	3
Safer et al., (2010)	3	1	3	2	1	2	3
Smith et al., (2006)	3	2	N/A	2	1	1	2
Smith et al., (2008)	3	2	N/A	2	1	2	2
Tapper et al. (2009)	3	1	3	1	1	1	3
Telch et al., (2000)	3	2	N/A	2	1	1	2
Telch et al., (2001)	3	1	3	2	1	2	3
Weineland et al., (2012a, 2012b)	2	1	3	2	1	2	2
Woolhouse et al., (2012)	3	2	N/A	2	1	2	2

EPHPP is Effective Public Health Practice Project Quality Assessment. Ratings are as follows: 1-Strong; 2-Moderate; 3-Weak. N/A means not applicable and was given to uncontrolled cohort studies that only had one group indicating that identifying and controlling for confounders across groups does not apply

### Interventions

#### Dialectical behavior therapy

Twenty-one of the 54 studies tested DBT as the MBI, with most providing in-person group therapy (n = 18). There are 4 main modules of DBT, including mindfulness (e.g., observing thoughts and emotions without judgment), distress tolerance (e.g., tolerating distressing emotions and internal sensations without engaging in maladaptive behaviors), emotion regulation (e.g., managing emotions more effectively through coping skills), and interpersonal effectiveness (e.g., practicing practical skills for emotional and mental well-being; Linehan, 2015). Eight studies employed DBT-BED (Safer et al., 2009), an adaptation of DBT specifically designed to address binge eating pathology by teaching healthy emotion regulation skills in place of binge eating (Klein et al., 2012; Lammers et al., 2020, 2022; Masson et al., 2013; Safer et al., 2010; Telch et al., 2000, 2001). Studies also adapted the standard DBT protocol to suit specific subsets of individuals with binge eating, such as: individuals undergoing joint treatment for BED or bulimia nervosa (BN) and borderline personality disorder (Chen et al., 2008), individuals seeking bariatric services (Mushquash & McMahan, 2015), and adolescents (Fischer & Peterson, 2015). Several studies explored condensed versions of DBT including Rahmani et al. (2018) which used a 20-session protocol based on Telch et al. (2001) and Linehan (2015). Blood et al. (2020) and Adams et al. (2021) followed the same manual (Safer et al., 2009), with Adams et al. (2021) condensing the treatment from 20 to 10 sessions, focusing on therapy-interfering behaviors, binge eating, mindless eating, and binge urges. Delparte et al. (2019) developed an 8-week online DBT skills group, and Braden et al. (2022) combined DBT and behavioral weight loss in 16 sessions. Conversely, some studies implemented more intensive DBT. Karam et al. (2022) described partial hospitalization programs offering "full-package DBT," including skills coaching, weekly groups, individual therapy, and consultation team meetings. Chen et al. (2017) offered an intensive 6-month program with weekly 2-h skills groups, 1-h individual therapy sessions, 2-h therapist consultation teams, and 24-h phone coaching. Researchers also focused on cultural and practical adaptations to improve accessibility. Cancian et al. (2019) translated and adapted DBT skills for Portuguese-speaking populations. Kamody et al. (2019) tailored their intervention for accessibility, offering multiple group options and staggered start dates. Two studies used a guided self-help program in which participants were given a manual to review on their own (Carter et al., 2020; Masson et al., 2013). In these guided self-help programs, participants either had the option to receive regular phone call support or video calls. As such, delivery of DBT in these studies was highly varied.

#### Acceptance and commitment therapy

Ten studies implemented Acceptance and Commitment Therapy (ACT). Across studies, the core components of ACT were consistently delivered, including cognitive defusion (the ability to see thoughts as mere internal events rather than facts), acceptance and mindfulness skills, addressing experiential avoidance, and focusing on values and committed action. While maintaining these fundamental elements, each study tailored its approach to address various aspects of eating behavior and weight management, such as coping with hunger and cravings, improving self-stigma and body image perceptions, and managing emotional eating. For example, Tapper et al. (2009) emphasized acceptance and mindfulness for emotional eating and cravings; Afari et al. (2019) focused on experiential avoidance in eating behaviors; Cuneo et al. (2018) highlighted the relationship between eating and stress; Lillis et al. (2011) combined psychoeducation, group activities, and individual mindfulness exercises to target feelings around body image, health values, and barriers to living in accordance with one’s values. Katterman et al. (2014) integrated ACT exercises with behavioral lifestyle changes, similar to Moffat et al. (2019) whose dietitians and psychologists jointly facilitated an ACT- based behavioral weight loss program. Another intervention provided ACT-based skills (values, acceptance, mindfulness, defusion, and committed action) for individuals after bariatric surgery, utilizing a combination of in-person sessions, online resources, telephone support, and recorded media (Weineland et al., 2012a, 2012b). Other web-based interventions included an ACT-influenced, internet CBT program (Strandskov et al., 2017); Boucher & colleagues (2016)’ “Mind, Body, Food” program designed to teach women intuitive eating skills; and Potts et al. (2022) offered phone coaching and virtual ACT coaching option.

#### Combined cognitive/behavioral and mindfulness-based approaches

Four studies explored combinations of mindfulness-based approaches with CBT for treating eating disorders. Baer et al. (2005b) modified an MBCT protocol (Segal et al., 2002) to emphasize non-judgmental awareness, recognizing thoughts as mental events, and developing adaptive coping strategies for binge eating. Woolhouse et al. (2012) introduced the Mindful Moderate Eating Group (MMEG), enhancing traditional elements of CBT for binge eating with mindfulness exercises. Leahey et al. (2008) tailored an MBCT protocol for individuals who had undergone bariatric surgery, focusing on encouraging self-monitoring practices, adjusting eating habits to align with post-surgical guidelines, and developing emotion regulation strategies. Courbasson et al. (2011) developed a novel intervention, Mindfulness-Action based Cognitive Behavioral Therapy (MACBT), for individuals struggling with both BED and substance use disorder. While varying in application and treatment duration, these interventions all maintained core components of CBT with the addition of mindfulness-based practices.

#### Mindfulness based stress reduction and adaptations

MBSR-based interventions, including meditation practice, breathing exercises, body scans, and gentle yoga, were used by two studies (Smith et al., 2006, 2008). Nine studies adapted MBSR protocol into interventions focused on mindful eating, particularly increasing awareness of eating patterns, improving emotional responses to negative affect, and making more conscious food choices while conducting guided meditations as seen in MBSR. These MBSR-based mindful eating programs emerged as a popular approach, including the Mindful Eating and Living program (MEAL; Dalen et al., 2010; Smith et al., 2018), and mindfulness-based eating awareness training (MB-EAT) interventions (Barnes & Kristeller, 2016; Kristeller & Hallett, 1999; Kristeller et al., 2014; Ugarte Perez et al., 2023; Wnuk et al., 2018). Salvo et al. (2022) and Minari et al. (2024) adapted MB-EAT for Brazilian populations, incorporating Brazilian dietary guidelines into the culinary workshops. Salvo & colleagues (2022) also randomized participants into a Mindfulness-Based Health Promotion program (MBHP), a national Brazilian program developed to promote healthy eating and enhanced quality of life. The MBHP protocol draws inspiration from MBSR (Kabat-Zinn, 2003), MBCT (Kuyken et al., 2010), Mindfulness-Based Relapse Prevention (MBRP; Hendershot et al., 2011), and mindfulness approaches from the UK-based Breathworks Institute (Cusens et al., 2010).

#### Other mindfulness-based interventions

Seven studies employed other mindfulness-based interventions. Like the studies above, these alternative interventions typically combined 2 or more treatment modalities. For instance, Brennan et al. (2020) incorporated Kripalu Yoga, a modern adaptation of Hatha yoga, emphasizing self-compassion and deepened awareness of bodily sensations throughout the structured classes. Carpenter et al. (2019) created the "Mind Your Weight" program, which integrated mindfulness practices with behavioral weight loss strategies. Corazon et al. (2018) developed a unique Nature-Based Therapy (NBT), combining elements from MBSR, ACT, and environmental psychology. This approach utilized a forest therapy garden setting to facilitate body and mind awareness exercises. Pinto-Gouveia et al. (2017) developed the BEfree program, integrating psychoeducation, mindfulness, and compassion-based components for women with binge eating and obesity. Duarte et al. (2017) modified the BEfree intervention to create the Compassionate Attention and Regulation of Eating (CARE) program, which incorporated parasympathetic vagal activation (soothing rhythm breathing) from Compassion-Focused Therapy (Gilbert, 2010; Gilbert & Choden, 2013) and mindful eating exercises from Goss’s overeating manual (Goss, 2011). Pepe et al. (2023) combined MB-EAT with a moderate energy restriction diet plan. Other integrative approaches included app-based mindfulness training, such as the Headspace® program used by Mercado et al. (2023) and Mensinger’s (2022) "No More Weighting" electronic course, which drew from mindful self-compassion science to address body trust and intuitive eating.

### Outcome measures

#### Eating Disorder Examination

Twenty-six studies (17 since the 2015 review) used the Eating Disorder Examination (EDE) to assess OBEs and diagnose BED (Fairburn & Cooper, 1993). The EDE is the current gold-standard assessment for eating pathology and behaviors, and it assesses the number of binge episodes and the number of days in which binge episodes occur. The EDE can be administered either as a clinical interview or as a self-report questionnaire (EDE-Q). Of the new studies included in this review, six used the clinical interview form of the EDE and 11 used the EDE-Q. One study used two questions adapted from the “screening version” of the EDE that dichotomously assessed the presence of binge eating and binge eating frequency over the last four weeks (Boucher et al., 2016). Two items (loss of control and guilt after eating) from the EDE were used by Leahey and colleagues (Leahey et al., 2008) to assess binge eating in a post-bariatric surgery sample. The other studies used the EDE assessed for BED. Most used the DSM-IV research criteria for BED (American Psychiatric Association, 1994) included in the EDE version 12 (Fairburn & Cooper, 1993) and later in version 16 of the EDE (Fairburn et al., 2008). Six of the studies also assessed BED with DSM-5 diagnostic criteria (American Psychiatric Association, 2013), which reduces the frequency of binge days to only once per week over the past 3 months and retains all other DSM-IV BED criteria.

#### Binge Eating Scale

Twenty-nine studies (21 new) used the Binge Eating Scale (BES), a 16-item self-report questionnaire assessing the severity of binge eating behavior in individuals with obesity (Gormally et al., 1982). The BES produces a severity score with ranges of 0–17 indicating no binge eating (none), 18–26 demonstrating moderate binge eating severity, and greater than 26 indicating severe binge eating. Tapper and colleagues (Tapper et al., 2009) used a shortened, 6-item version of the scale assessing the central symptoms of binge eating, which they reported had good internal reliability at baseline in their sample (alpha = 0.74).

#### Other measures of binge eating

Three studies assessed binge eating with self-reported number of weekly binges by diary card (Klein et al., 2012), phone, or in person during treatment sessions (Kristeller & Hallett, 1999; Lillis et al., 2011). Less commonly used measures of binge eating included the Eating Disorders Inventory-3 symptom checklist (EDI-3 SC; Garner, 2004), the Multifactorial Assessment of Eating Disorders Scale (MAEDS; Anderson et al., 1999), the Disordered Eating after Bariatric Surgery (DEBS) questionnaire (Weineland et al., 2012a, 2012b), and the Questionnaire on Eating and Weight Patterns-5 (QWEP-5; Yanovski et al., 2015). One study assessed the percentage of participants who binged using the EDI-3 SC and the binge eating subscale of the MAEDS (Woolhouse et al., 2012). Weineland and colleagues (Weineland et al., 2012a, 2012b) used the DEBS, formerly called the Subjective Binge Eating Questionnaire for Bariatric Surgery Patients, which is a self-report measure they developed for assessing binge eating behavior in a post-bariatric surgery population that they report had reasonable psychometric properties (Weineland et al., 2012a, 2012b).

### Effects sizes and meta-analysis

Effect sizes were calculated or extracted from 52 studies. Two studies did not provide sufficient data for effect size calculation (Baer et al., 2005b; Kamody et al., 2019). The 52 studies yielded 90 within-group effect sizes, 35 between-group effect sizes comparing MBIs with control groups that did not contain psychological interventions, and 29 between-group effect sizes comparing MBIs with psychological interventions. Using 0.2, 0.5, and 0.8 to interpret small, medium, and large effects (Cohen, 1992), 54 of the 90 within-group effect sizes were large, 23 were medium (including 1 negative effect size, indicating an increase of binge eating after receiving MBIs (Katterman et al., 2014)), 10 were small, and the remaining 3 were negligible. For studies comparing MBIs with control groups without psychological interventions, 18 out of the 35 effect sizes were large, 2 were medium, 5 were small in favor of MBIs, indicating MBIs demonstrated larger effects in reducing binge eating than the control groups, and 1 small in favor of the control group. For studies comparing MBIs with control groups containing psychological interventions, 3 of the 29 effect sizes were medium (2 in favor of MBIs, 1 in favor of the control group—MEAL without mindfulness components), 13 were small (7 in favor of MBIs, 6 in favor of the control groups—3 CBTs, 1 BWL + CBT, 1 MEAL without mindfulness components).

**Fig. 2:**
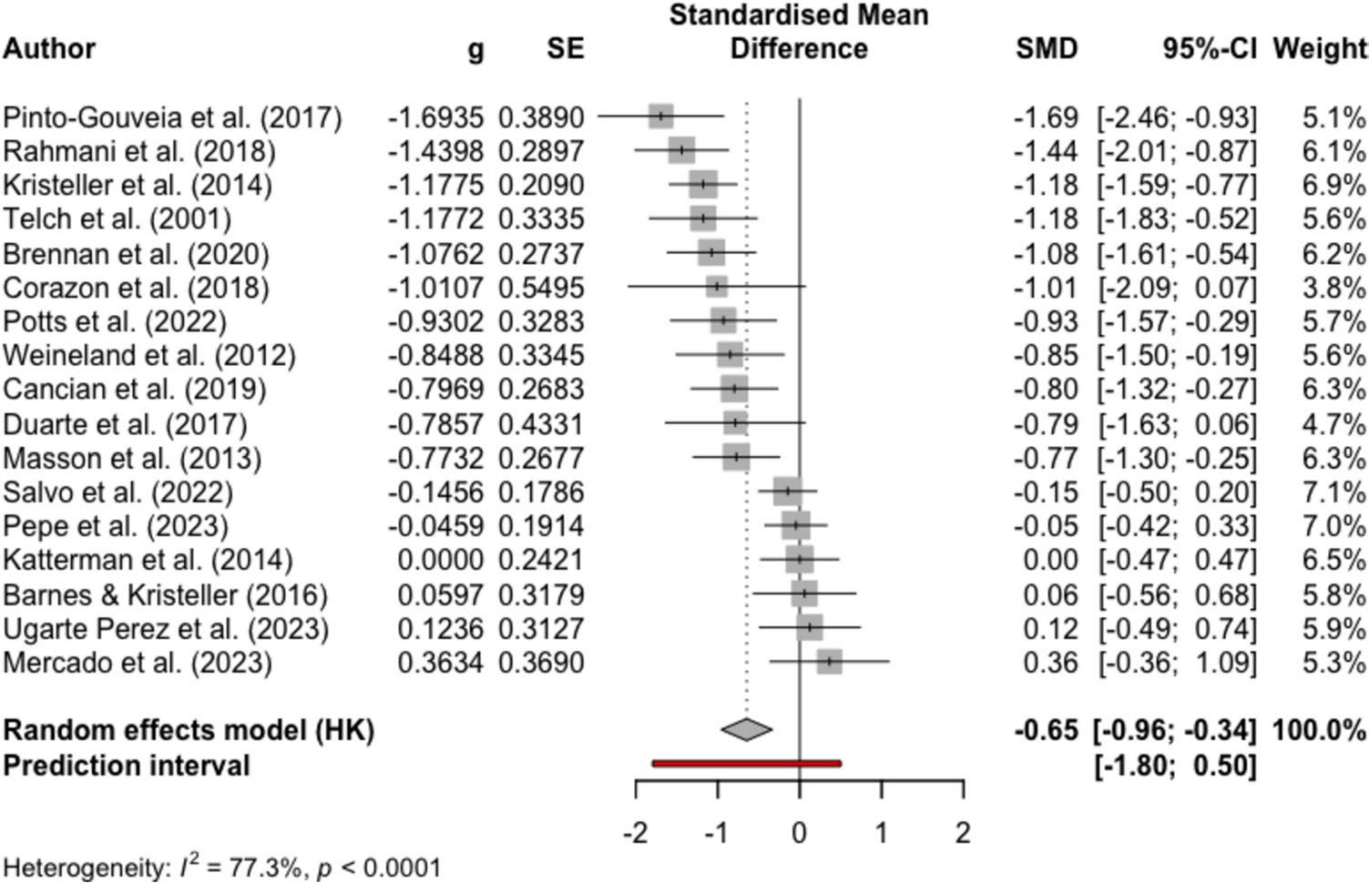
Forest plot for meta-analysis comparing MBIs with non-psychological intervention controls at the end of treatment

**Fig. 3:**
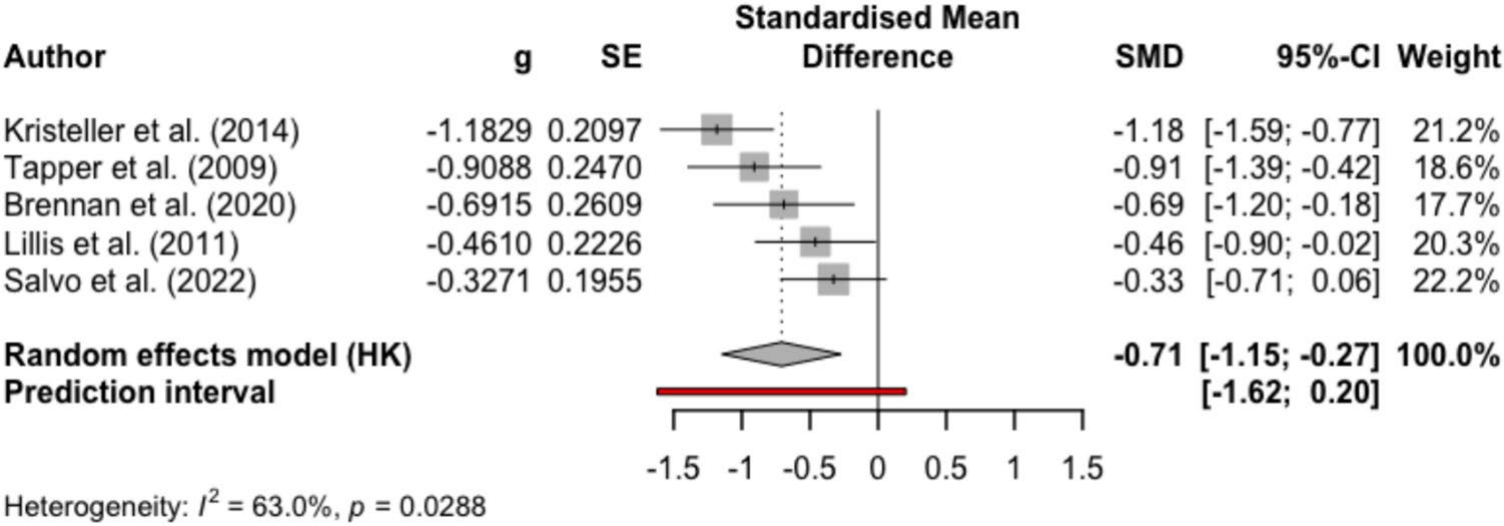
Forest plot for meta-analysis comparing MBIs with non-psychological intervention controls at follow up

**Fig. 4:**
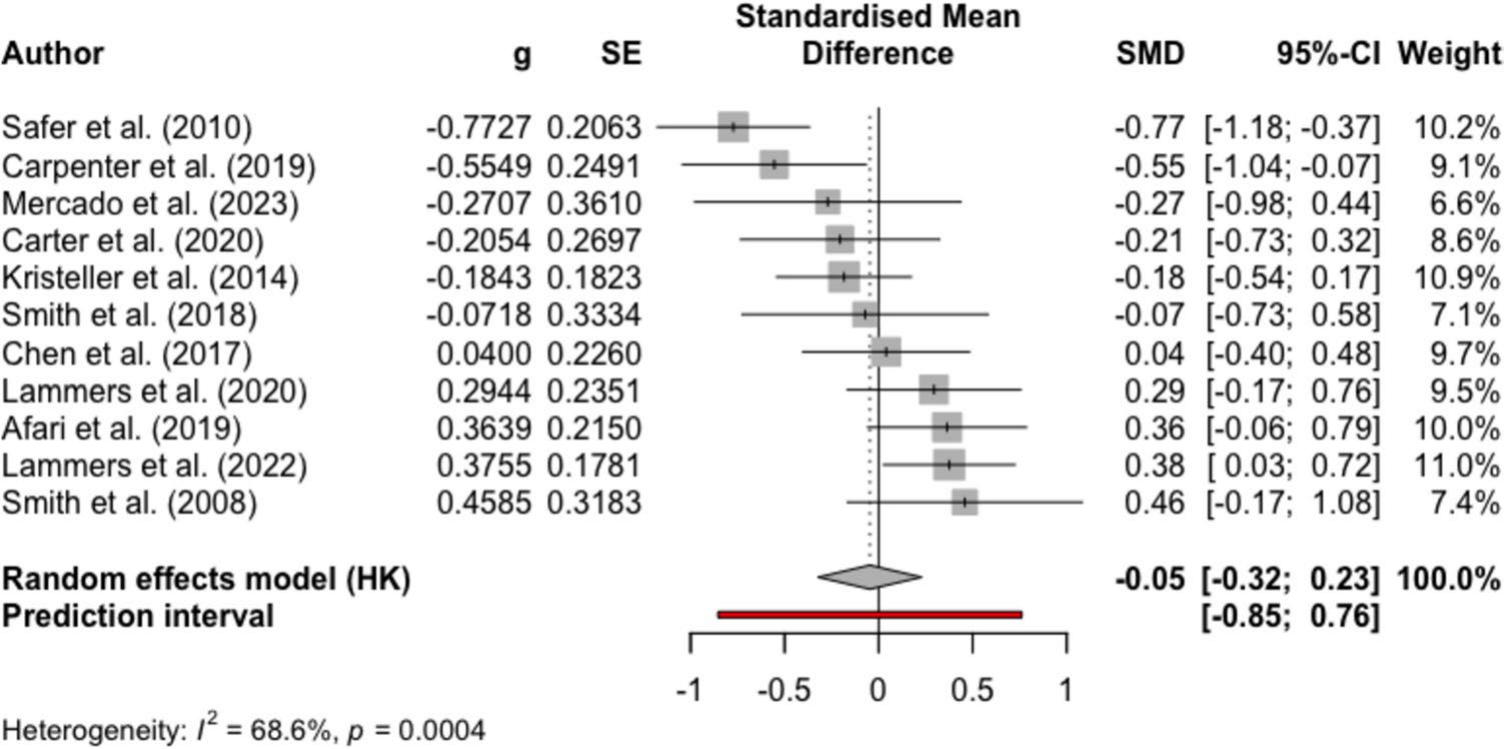
Forest plot for meta-analysis comparing MBIs with psychological intervention controls at the end of treatment

**Fig. 5:**
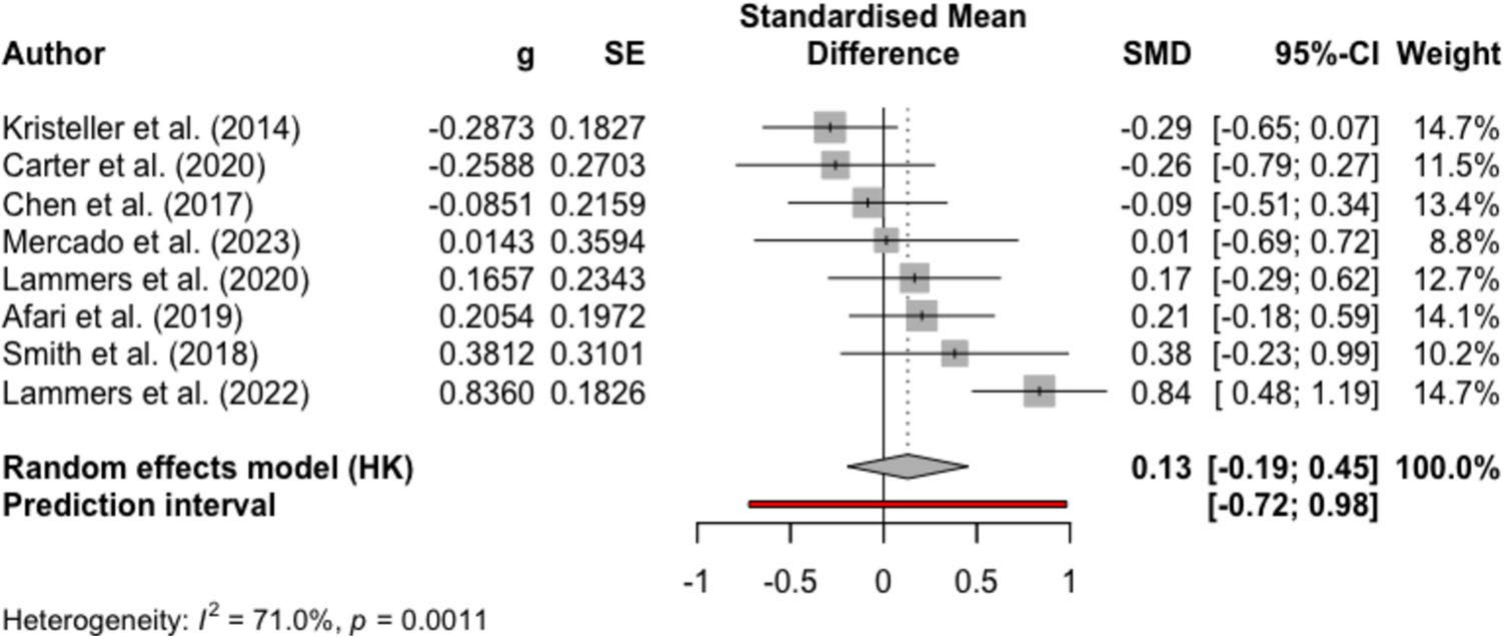
Forest plot for meta-analysis comparing MBIs with psychological intervention controls at follow-up

Figures 2, 3, 4, and 5 present the effect sizes and the forest plots for the studies included in the between-group random effects meta-analysis for MBIs versus non-psychological controls and psychological controls at EOT and FU, respectively. Results from the meta-analysis supported medium-large effects of MBIs compared to non-psychological controls in reducing binge eating at EOT (mean Hedge’s *g* = − 0.65, 95% CI − 0.96, − 0.34, *p* < 0.001, *k* = 17) and at FU (mean Hedge’s *g* = − 0.71, 95% CI − 1.15, − 0.27, *p* = 0.01, *k* = 5). Results also indicated substantial heterogeneity among studies (EOT *I*^*2*^ = 77.3%; FU *I*^*2*^ = 63.0%). For the comparisons between MBIs and psychological controls, the result from the meta-analysis yielded negligible effect sizes for MBIs at EOT (mean Hedge’s *g* = − 0.05, 95% CI − 0.32, 0.23, *p* = 0.71, *k* = 11) and at FU (mean Hedge’s *g* = 0.13, 95% CI − 0.19, 0.45, *p* = 0.37, *k* = 8). The results comparing MBIs with psychological controls also suggested substantial heterogeneity among studies (EOT *I*^*2*^ = 68.6%; FU *I*^*2*^ = 71.0%), and therefore, the results of the meta-analyses should be interpreted with caution.

#### By interventions

Ten studies that examined ACT yielded 14 within-group effect sizes and 12 between-group effect sizes. One of the 14 within-group effect sizes was large, 7 were medium (with 2 negative medium effect sizes from Katterman et al., 2014), and 5 were small. Four of the 12 between-group effect sizes were large, 1 was medium, and 1 was small, all in favor of ACT. Two of the between-group effect sizes were small, both in favor of the control group (BWL + CBT), indicating the control group had a larger effect in reducing binge eating compared to ACT. For DBT studies, all 37 within-group effect sizes were either medium (n = 10) or large (n = 27). Of the 22 between-group effect sizes DBT studies yielded, 5 of them were large, 2 were medium, 5 were small (3 in favor of DBT, 2 in favor of the control group—CBT), and the remaining 10 studies yielded negligible effect sizes. The remaining 22 studies examined other types of MBIs and yielded 41 within-group effect sizes, with 26 large, 8 medium, 5 small, and 2 negligible, and 32 between-group effect sizes, with 11 large, 3 medium (2 in favor of MBIs, 1 in favor of the control group—MEAL without mindfulness components), 11 small (8 in favor of MBIs, 3 in favor of the control groups—MEAL without mindfulness components, CBT, and WL), and 7 negligible.

#### By treatment target

Thirty-two studies examined interventions that directly targeted binge eating. Of the 60 within-group effect sizes these studies reported, 42 were large, 14 were medium, 2 were small, and 2 were negligible. For between-group effect sizes, 16 of the 41 effect sizes were large, 3 were medium, 7 were small in favor of MBIs, and 11 were negligible. Interventions from the remaining studies targeted other health outcomes, such as weight loss and healthy eating, but measured binge eating as a secondary outcome. These studies yielded 30 within-group effect sizes (12 large, 9 medium, 8 small, and 1 negligible) and 23 between-group effect sizes (2 large, 3 medium with 2 in favor of MBIs and 1 in favor of the control group, 8 small with 5 in favor of MBIs and 3 in favor of the control group, and 10 negligible).

#### By data collection methods

Eleven studies used a clinical interview (EDE) to collect binge eating data from participants, and some studies reported more than one variable within the EDE (e.g., the number of OBEs and OBDs). These 11 studies yielded 26 within-group effect sizes, including 21 large and 5 medium, and 20 between-group effect sizes, including 6 large, 2 medium, 5 small (4 in favor of MBIs, 1 in favor of the control group), and 7 negligible. Forty-five studies used self-reported questionnaires for data collection and reported 64 within-group effect sizes (33 large, 18 medium, 10 small, and 3 negligible) and 44 between-group effect sizes (12 large, 4 medium with 3 in favor of MBIs and 1 in favor the control group, 14 small with 8 in favor of MBIs and 6 in favor of the control groups, and 14 negligible).

### Update from the original review

Thirty-five new studies were added in this updated review, compared to 19 in the original review, indicating that MBI research has gained popularity in recent years. For these newly added studies, similar proportion of the effect sizes were medium or large (within: 84.21%; between (with control groups that did not contain active psychological interventions): 52.63%) compared to the original review by Godfrey & colleagues (2015; within: 81.82%; between: 60%). More recent studies were conducted outside of the US (n = 23) by more researchers and teams. Recent studies also were more likely to recruit participants with overweight and obesity (n = 17; 48.57%) than studies in the original review (n = 2; 10.53%). Additionally, researchers have started to examine the effectiveness of MBIs in adolescent populations (n = 3 compared to 0 in the previous review). Moreover, self-help interventions (n = 4; 2 in the previous review), including both guided and unguided versions, have been explored more, as well as technology-based intervention components (n = 11; 2 in the previous review), such as phone coaching and app-based intervention. The more recent studies also utilized novel techniques and combinations of multiple innovative intervention strategies (e.g., Kripalu Yoga, nature-based therapy, etc.; Brennan et al., 2020; Corazon et al., 2018) to come up with unique integrative MBIs. Regarding follow-up period, a limitation mentioned in the original review, approximately the same proportion of the new studies followed-up with participants after treatment ended (40.00%; original review: 42.11%). Most studies had follow-up periods up to 6 months post-treatment, one study had 9 months, and one had 12 months, which was also comparable to the original review.

## Discussion

### Overall findings

This updated systematic review examined the effects of MBIs on binge eating by reviewing evidence from 54 unique studies, including 35 new studies published between 2014 and 2024 and 19 studies from the original review (Godfrey et al., 2015). DBT was the most common intervention approach, followed by ACT and MBSR. Twenty-two studies recruited participants with binge eating, 19 required participants with BMI in the overweight and/or obese range, and 5 had bariatric samples. Binge eating was measured using clinical interviews (e.g., EDE) and self-reported questionnaires (e.g., EDE-Q, BES). The majority of the studies showed large within-group and medium-large between-group (with non-psychological intervention control groups) effect sizes. The between-group random effects meta-analysis results demonstrated medium-large effect sizes for MBIs relative to non-psychological intervention controls at EOT and FU, suggesting that MBIs showed medium to large effects on reducing binge eating, and thus could be considered effective. However, when comparing MBIs with psychological intervention controls, the meta-analysis yielded negligible effects for MBIs at EOT and FU, indicating that MBIs and other psychological interventions are comparable in reducing binge eating. The high level of heterogeneity among studies could potentially explain the negligible effect sizes comparing MBIs and active controls, as the studies examined various types of interventions (both for MBIs and the active controls) and used different outcome measures (e.g., self-report vs clinical interview). Therefore, future research should examine the effectiveness of specific types and components of MBIs, and compare MBIs with specific types of active control groups. The findings align with findings from previous reviews on MBIs for binge eating (Godfrey et al., 2015; Grohmann & Laws, 2021). The quality of the newly added studies included in this review was relatively higher compared to the studies in the original review, as a greater percentage of the newly-added studies received a strong rating than the original review. However, the overall quality of the newly included studies was still moderate, which has not changed since the original review.

Studies that tested DBT tended to have larger effect sizes compared to other types of interventions. One potential explanation could be that a majority of the DBT interventions in this review were relatively more intensive regarding session duration, session modality, and treatment settings compared to other MBIs. Eleven of the 21 DBT interventions comprised 20 or more sessions, and another DBT study was conducted in higher-level care settings. For session lengths, 14 of these interventions lasted for 2 h or more for each session. Additionally, 4 of the DBT interventions included both individual and group therapy (Chen et al., 2008, 2017; Fischer & Peterson, 2015; Karam et al., 2022), and none of the other types of interventions had such a mixture of treatment modalities. Indeed, a previous meta-analysis on the efficacy of different types of treatment for BED suggested that psychotherapy might be more efficacious when provided with greater intensity (Hilbert et al., 2019). Future research should compare DBT to comparison groups that are matched in intensity/modality, and compare DBT to other MBIs.

The results also showed that MBIs specifically designed to target binge eating had larger effects in reducing binge eating than MBIs primarily targeted other health outcomes (e.g., weight loss). Studies with MBIs directly targeting binge eating were more likely to require participants to engage in at least a certain amount of binge episodes as an inclusion criterion, which could potentially explain the higher effectiveness of these interventions. Studies targeting other behaviors and measuring binge eating as a secondary outcome might experience a floor effect because of the low baseline binge eating. MBIs directly targeting binge eating might also include more active components and dosages that are dedicated to addressing the maintaining factors of binge eating. For example, almost all DBT studies that had binge eating as the core intervention target used a modified version of DBT that was adapted for this specific population (Safer et al., 2009), and MB-EAT programs focusing on binge eating also devoted a large proportion of the intervention contents to identifying and managing binge triggers (Kristeller et al., 2014). However, it remains unclear whether it was the mindfulness-related strategies (e.g., mindfulness practice when experiencing urges), or other intervention components (e.g., identifying triggers for binge eating), or the combination of them that was the active ingredient that contributed to the reduction of binge eating. Therefore, future studies should attempt to identify the most effective intervention components.

The current review also found that more studies that used a clinical interview (EDE) to measure binge eating (instead of a self-report measure) yielded medium and large effect sizes compared to studies that used self-report questionnaires. The EDE requires a trained assessor to ask a comprehensive list of questions to determine OBE ratings (e.g., specific food items consumed during the episode, feeling of cannot stop eating once started and/or even if wanted to) and also provide detailed explanations of constructs that participants might be confused about (e.g., explaining loss of control as a feeling of ball rolling down a hill or a train going off the track), and therefore the ratings might be less likely to be influenced by participants’ subjective interpretations. Self-report questionnaires typically rate OBEs by only asking participants to self-determine whether they thought the amount of food they consumed was objectively large and whether they experienced loss of control feeling, which could be easily impacted by participants’ understanding of the constructs and their recall biases (Everett et al., 2021). Indeed, previous research has found discrepancies in ratings between clinical interviews and self-report questionnaires, especially on more complex constructs like binge eating, especially in individuals with overweight/obesity (Everett et al., 2021; Fairburn & Beglin, 1994), and has also suggested that self-report questionnaires should clarify the definitions of complex features (e.g., binge episodes) to improve the consistency in participants’ responses (Wilfley et al., 1997). Therefore, participants’ varying levels of comprehension of the constructs assessed using self-report questionnaires might also contribute to the difference observed in this review.

Since the original review by Godfrey et al. (2015), the number of studies on MBIs for binge eating has steadily increased, and these studies were from various research groups around the world, indicating that this area of research is still growing worldwide. The new studies recruited more participants with overweight and obesity. Overweight and obesity are common comorbid health conditions for patients with binge eating, and weight loss interventions often have overlapping components with binge eating interventions. As researchers started to explore integrative MBIs that combined intervention strategies from different approaches (e.g., Corazon & colleagues’ NBT (2018) combining ACT and MBSR, Pinto-Gouveia et al.’s BEfree (2017) combining mindfulness practices and compassion-focused strategies), future research could also consider developing and testing novel integrative MBIs with effective intervention components to target patients with both binge eating and obesity. Additionally, the follow-up period was identified as a limitation by both of the previous reviews (Godfrey et al., 2015; Grohmann & Laws, 2021). The same limitation remains for this updated review, as roughly the same proportion of the studies followed up with participants after treatment ended compared to the original review, and the length of the follow-up period was also relatively short. Thus, future studies should conduct more follow-up assessments to better evaluate the long-term effectiveness of MBIs.

### Limitations and future directions

There were several limitations for this review. First, the quality of evidence was limited by not including covariates in analyses, selection bias, and withdrawal rate (e.g., not reporting how many or why participants dropped out of the study). These weaknesses should be addressed in future research to strengthen available evidence. For example, measuring outcomes of interventions could be significantly influenced by the presence of confounders and should therefore be controlled. Additionally, the substantial differences in methodology and statistical analyses mentioned in the original review still remained a limitation in the current review. Although more studies required participants to engage in binge eating prior to treatment, there were still variations in levels of binge eating severity among different samples. There were also diverse types of interventions with various intervention targets. Although these differences in methodology and statistical analyses might influence the results of this systematic review, they are still important to recognize as they also reflect and represent the real-world variety of patient populations, intervention focuses, and measures employed with different types of MBIs.

The current review also has crucial implications for future research on MBIs for binge eating. Although the number of RCTs has been growing, more RCTs are still needed to compare MBIs with both no-intervention control groups, as well as with other psychological interventions, like standard CBT for binge eating (Fairburn et al., 2009). Seven studies in the current review compared MBIs with CBT or interventions with CBT components, and only 4 of them were RCTs (Afari et al., 2019; Carpenter et al., 2019; Chen et al., 2017; Kristeller et al., 2014). As components of MBIs became more diverse and more integrative interventions emerged, RCTs comparing MBIs with CBT could help researchers and clinicians identify the most effective components of these interventions and continue to optimize treatment outcomes. Similarly, future research could conduct meta-analyses to quantitatively examine the relative effectiveness of different types of MBIs (e.g., ACT vs MB-EAT), intervention delivery methods (e.g., in-person vs app-based interventions), and treatment targets (e.g., binge eating vs emotional eating).

## Conclusion

This updated systematic review found that MBIs continued to demonstrate large or medium-large effects in reducing binge eating. Among all types of MBIs included in this review, DBT demonstrated larger effects compared to other MBIs. MBIs appeared to be more effective if binge eating was the direct intervention target. Additionally, studies that used clinical interviews to collect binge eating outcome data tended to show larger effects. Future research should follow up with participants for a longer period of time after treatment ends, conduct more RCTs comparing MBIs with other psychological interventions, and quantitatively examine the effectiveness of different types of MBIs, intervention delivery methods, and intervention targets.

## Data Availability

The data of this study are available from the corresponding author upon reasonable request.
